# First passage events in biological systems with non-exponential inter-event times

**DOI:** 10.1038/s41598-018-32961-7

**Published:** 2018-10-10

**Authors:** Mario Castro, Martín López-García, Grant Lythe, Carmen Molina-París

**Affiliations:** 1 Grupo Interdisciplinar de Sistemas Complejos (GISC) and DNL, Universidad Pontificia Comillas, Madrid, E-28015 Spain; 2 Department of Applied Mathematics, School of Mathematics, University of Leeds, Leeds, LS2 9JT UK

## Abstract

It is often possible to model the dynamics of biological systems as a series of discrete transitions between a finite set of observable states (or *compartments*). When the residence times in each state, or inter-event times more generally, are exponentially distributed, then one can write a set of ordinary differential equations, which accurately describe the evolution of mean quantities. Non-exponential inter-event times can also be experimentally observed, but are more difficult to analyse mathematically. In this paper, we focus on the computation of first passage events and their probabilities in biological systems with non-exponential inter-event times. We show, with three case studies from Molecular Immunology, Virology and Epidemiology, that significant errors are introduced when drawing conclusions based on the assumption that inter-event times are exponentially distributed. Our approach allows these errors to be avoided with the use of phase-type distributions that approximate arbitrarily distributed inter-event times.

## Introduction

Biology provides many instances of processes where dynamics far from equilibrium determines the fate of the system. Notable examples include the ability of a virus to infect a cell and produce new virions before the immune system detects it, or the initiation of a signalling cascade after ligand-receptor binding and phosphorylation events^1^. Many mathematical models of these biological systems have been developed at the population level and make use of ordinary differential equations. However, new experimental techniques that allow study of single individual agents (cells, receptors, genes, …) demand stochastic descriptions^2^ of these biological processes. Stochastic methods are growing in popularity, partly driven by new questions posed by more sophisticated experiments, but also by the increasing capacity of modern computers. The common Markovian assumption in stochastic models is that events in the future, given information only about the present state of the system, are independent of those that occurred in the past (the so-called memoryless property). A particular property of continuous time Markov processes with discrete state spaces (*i*.*e*., continuous time Markov chains (CTMCs)) is that the time for each event to occur is exponentially distributed. Thus, the next event to occur in the CTMC is the result of a competition between exponentially-distributed *absolute waiting times*, leading to the Gillespie algorithm^3^ for their efficient numerical simulation.

A stochastic process in which inter-event times are not exponentially distributed is not strictly Markovian because, as well as depending on the current state of the system, the choice and timing of the next event depend on how long the process has been in its current state. It is not difficult to find experimental situations where the Markovian assumption is too strong. For instance, the probability density of the time from infection of a cell until the same cell produces a new virion is not exponentially distributed^4^; multiple intermediate steps (virus entry, migration to the nucleus, hijacking of the cellular machinery, viral assembly, …) take place inside the cell between these two events. Another biological process where it has been suggested that the Markovian assumption might be too strong is cell proliferation^5^, where Erlang distributions for modelling the inter-event times within the cell cycle might be more appropriate than the exponential distribution^5^. It is also important to keep in mind that, when more than one transition is possible out of a given state, experimentally observed distributions of times for a particular transition to occur are always conditioned (also referred to as censored^6^) on the chosen transition actually taking place.

**Figure 1:**
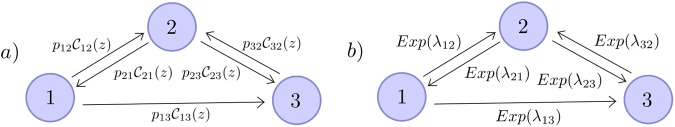
(**a**) Flowgraph representation of a continuous time semi-Markov process, ${\mathscr{X}}$, defined on ${\mathscr{S}}=\{1,2,3\}$. (**b**) Markovian version of the process ${\mathscr{X}}$, where all events $i\to j$, with *i*, $j\in {\mathscr{S}}$, occur with exponentially distributed times ${T}_{i\to j}\sim Exp({\lambda }_{ij})$.

In this paper, we relax the Markovian assumption by requiring that the process is Markovian only at arrival times: once the process jumps into any state, the future of the system does not depend on its past, but only on how long the process has been in that state, leading to a *semi-Markov* process^7^. Although Markov processes have been widely studied in the literature, and their applicability has been shown in a wide range of areas such as Biology, Epidemiology, Immunology, Population Dynamics or Queuing Theory, semi-Markov processes have mostly been treated as an advanced and theoretical topic with a focus on definitions and limit theorems^8^, but with limited applications^9,10^. A practical way of analysing semi-Markov processes, and linking them to real data, is the “flowgraph” methodology^8^. Flowgraphs are elegant graphical representations of semi-Markov processes: the system is depicted as a directed network formed by the possible states of the process, where arrows represent possible events (or transitions) causing the process to move (or *jump*) from its present state to another (see Fig. 1a). Each event has an associated probability and conditioned waiting time. It is convenient to describe these conditioned waiting times by their moment generating functions (or Laplace-Stieltjes transforms), as in Fig. 1a. One of the advantages of flowgraphs is that they bypass the required mathematical characterisation of semi-Markov processes, since they are designed to handle real data^8^.

The aim when analysing flowgraph models is usually to study probabilities of potential outcomes (*e*.*g*., the probability that the process in Fig. 1a reaches state 3 before reaching state 2, if the process starts at 1), or first passage times (*e*.*g*., the time for the process to reach state 3, if the process starts at 1). In this paper, we focus on computing a first passage time (the time for the process to reach a given state) when some inter-event times are not exponentially distributed. First passage times have been widely analysed in the literature when considering stochastic systems with continuous states and with specific applications in Biology^11,12^. In this manuscript, we focus on computing first passage times in systems with discrete states. For discrete states, first passage times have been successfully analysed in the Markovian case^13^ [Chapter 1], and some work has already been carried out within the theory of semi-Markov processes but under very restrictive assumptions^14^ and with a strong theoretical focus. When considering the flowgraph methodology as a way of analysing semi-Markov processes, first passage times can be analysed but it is usual to assume that the probabilities of each event occurring in the system, as well as the conditioned waiting time distributions, are known^8^.

The purpose of this paper is threefold:

1. to obtain the relationship between absolute and conditioned waiting times when all or some inter-event times are not exponentially distributed,
2. to introduce a methodology based on phase-type distributions to approximate non-Markovian events^13^, while maintaining analytical tractability, and
3. to show that considering (*e*.*g*., biological) processes with the inappropriate assumption that all inter-event times are exponentially distributed can lead to incorrect predictions (*e*.*g*., when analysing first passage times).

We introduce our results using three case studies from different biological areas. We summarise the main mathematical results in the Materials and Methods section, and devote most of the Results section, to illustrating their applicability in three examples taken from Immunobiology (at the receptor level), Virology (at the cell level) and Epidemiology (at the host level). Further details of the derivation and additional results may be found in the Supplementary Information.

## Materials and Methods

### The flowgraph methodology

We consider a continuous time stochastic process ${\mathscr{X}}=\{X(t):t\ge 0\}$ defined over a finite and discrete space of states, ${\mathscr{S}}$. Given any state $i\in {\mathscr{S}}$, we define $A(i)=\{{i}_{1},{i}_{2},\ldots ,{i}_{{n}_{i}}\}$ to be the set of states directly accessible (*i*.*e*., in one *jump*) from *i*, where ${n}_{i}$ is the cardinality of $A(i)$. For example, for the process ${\mathscr{X}}$ in Fig. 1, we have $A(1)=\{2,3\}$, $A(2)=\{1,3\}$ and $A(3)=\{2\}$.

If the process ${\mathscr{X}}$ starts at $t=0$ in state $i\in {\mathscr{S}}$, $X(0)=i$, it jumps from *i* to any other state $j\in A(i)$ after a random time. This event (or transition) $i\to j$ actually occurs, if and only if, the *absolute waiting time* ${T}_{i\to j}$ for this event is shorter than any other *competing* absolute waiting time ${T}_{i\to j^{\prime} }$, $j^{\prime} \in A(i)\backslash \{j\}$. This means that the transition probability of the event $i\to j$ can be written as

$${p}_{ij}={\mathbb{P}}(i\to j)={\mathbb{P}}({T}_{i\to j} < {T}_{i\to j^{\prime} },\,\forall j^{\prime} \in A(i)\backslash \{j\}),\,j\in A(i).$$

The time for this transition to take place, conditioned on actually occurring, ${T}_{i\to j}|i\to j$ (*i*.*e*., the *conditioned waiting time*), can be described in terms of its Laplace-Stieltjes transform

$${{\mathscr{C}}}_{ij}(z)=E[{e}^{-z{T}_{i\to j}}|i\to j].$$

We assume that the process is Markovian at arrival instants. Namely, after the arrival at a given state of the process $i\in {\mathscr{S}}$, future transitions do not depend on the past history of the process. However, we note that once the process has been in a given state for some time, its future evolution might, in fact, depend on how long the process has been in that state, so that the process is only Markovian at arrival to any given state, and is not a Markov process, but a semi-Markov process^7^. A flowgraph representation^8^ [Chapter 2] of ${\mathscr{X}}$ is a set of nodes (*states*) connected by arrows that represent the possible transitions between states. The arrows are labelled with transition probabilities, ${p}_{ij}$, and conditioned Laplace-Stieltjes transforms, ${{\mathscr{C}}}_{ij}(z)$, as defined above (see the example in Fig. 1a). We note that the Laplace-Stieltjes transforms can be replaced by the moment generating functions, ${M}_{ij}(s)=E[{e}^{s{T}_{i\to j}}|i\to j]={{\mathscr{C}}}_{ij}(\,-\,s)$ for *i*, $j\in {\mathscr{S}}$. Below, we will use the notation of Fig. 1b, where each arrow is accompanied by its absolute waiting time distribution. The reader can translate between both notations using Fig. 1.

Let us imagine that the process ${\mathscr{X}}$ is defined on the space of states ${\mathscr{S}}=\{1,2,\ldots ,N\}\cup \{{\rm{\Omega }}\}$, with initial state $X(0)=i\in {\mathscr{S}}$, and that the interest is in studying the first passage time to state ${\rm{\Omega }}$, that is, ${T}_{i}({\rm{\Omega }})={\rm{\inf }}\{t\ge 0:X(t)={\rm{\Omega }}\}$. This time can be analysed in terms of its Laplace-Stieltjes transform, ${ {\mathcal L} }_{i}(z)=E[{e}^{-z{T}_{i}({\rm{\Omega }})}]$, which can be computed by carrying out a first step argument (we can assume that, under the semi-Markov property, only the current state affects the next transition), as follows:

$$\begin{array}{rcl}{ {\mathcal L} }_{i}(z) & = & \sum _{j\in A(i)}\,E[{e}^{-z{T}_{i}({\rm{\Omega }})}|i\to j]\,{\mathbb{P}}(i\to j)\\  & = & \sum _{j\in A(i)}\,E[{e}^{-z({T}_{i\to j}+{T}_{j}({\rm{\Omega }}))}|i\to j]\,{\mathbb{P}}(i\to j)\\  & = & \sum _{j\in A(i)}\,E[{e}^{-z{T}_{i\to j}}|i\to j]E[{e}^{-z{T}_{j}({\rm{\Omega }})}|i\to j]\,{\mathbb{P}}(i\to j)\\  & = & \sum _{j\in A(i)}\,{p}_{ij}\,{{\mathscr{C}}}_{ij}(z)\,{ {\mathcal L} }_{j}(z),\end{array}$$

which leads to the following system of equations in matrix form^9^

1 $$\mathop{(\begin{array}{ccccc}1 & -{p}_{12}{{\mathscr{C}}}_{12}(z) & \ldots  & -{p}_{1N}{{\mathscr{C}}}_{1N}(z) & -{p}_{1{\rm{\Omega }}}{{\mathscr{C}}}_{1{\rm{\Omega }}}(z)\\ -{p}_{21}{{\mathscr{C}}}_{21}(z) & 1 & \ldots  & -{p}_{2N}{{\mathscr{C}}}_{2N}(z) & -{p}_{2{\rm{\Omega }}}{{\mathscr{C}}}_{2{\rm{\Omega }}}(z)\\ \vdots  & \vdots  & \ddots  & \vdots  & \vdots \\ -{p}_{N1}{{\mathscr{C}}}_{N1}(z) & -{p}_{N2}{{\mathscr{C}}}_{N2}(z) & \ldots  & 1 & -{p}_{N{\rm{\Omega }}}{{\mathscr{C}}}_{N{\rm{\Omega }}}(z)\\ 0 & 0 & \ldots  & \ldots  & 1\end{array})}\limits_{{\bf{A}}(z)}\,(\begin{array}{c}{{\mathscr{L}}}_{1}(z)\\ {{\mathscr{L}}}_{2}(z)\\ \vdots \\ {{\mathscr{L}}}_{N}(z)\\ {{\mathscr{L}}}_{{\rm{\Omega }}}(z)\end{array})=\mathop{(\begin{array}{c}0\\ 0\\ \vdots \\ 0\\ 1\end{array})}\limits_{{\bf{b}}(z)},$$

with ${ {\mathcal L} }_{{\rm{\Omega }}}(z)\equiv 1$ (as the time to reach ${\rm{\Omega }}$ starting at ${\rm{\Omega }}$ is 0). Once the Laplace-Stieltjes transform ${ {\mathcal L} }_{i}(z)$ has been determined for the particular initial state of interest $i\in {\mathscr{S}}$, the moments of ${T}_{i}({\rm{\Omega }})$ can be calculated as follows

$${\mu }_{i}^{(k)}\equiv E[{T}_{i}{({\rm{\Omega }})}^{k}]={(-1)}^{k}{\frac{{d}^{k}{{\mathscr{L}}}_{i}(z)}{d{z}^{k}}|}_{z=0},$$

and the density function of ${T}_{i}({\rm{\Omega }})$ can be approximated by a numerical inversion of the Laplace-Stieltjes transform ${ {\mathcal L} }_{i}(z)$^15^.

**Figure 2:**
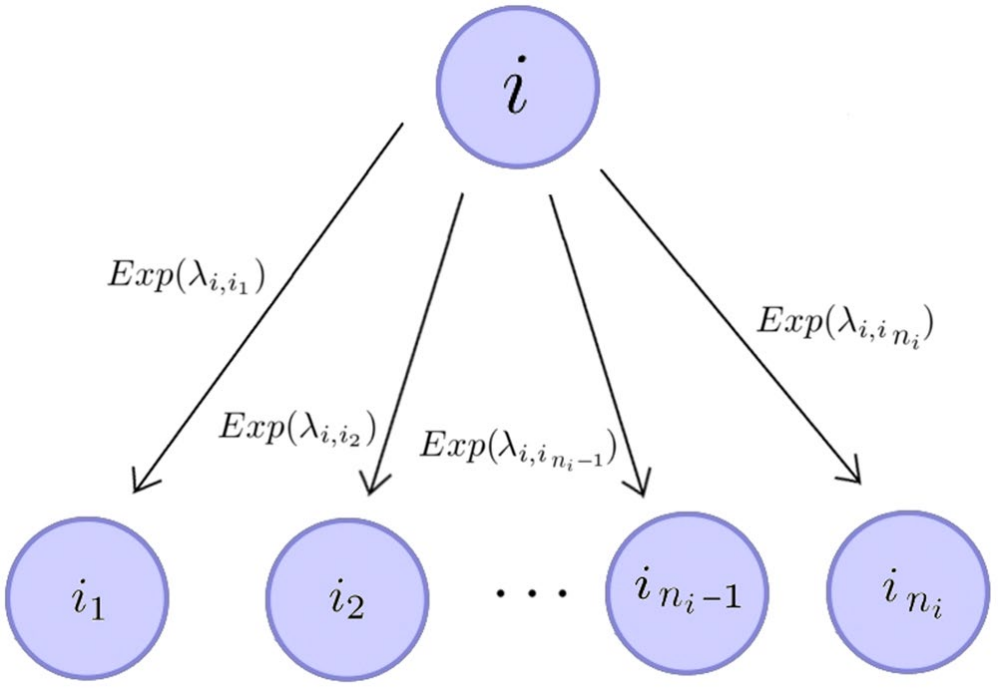
From any state $i\in {\mathscr{S}}$, the absolute waiting times to any state $j\in A(i)=\{{i}_{1},{i}_{2},\ldots ,{i}_{{n}_{i}}\}$ are exponentially distributed, ${T}_{i\to j}\sim Exp({\lambda }_{ij})$, leading to a process such as the one described in Fig. 1b.

We note that the applicability of Eq. (1) relies on the knowledge of (or estimations from experimental data) the (competing) probabilities ${p}_{ij}$ and the conditioned waiting time transforms ${{\mathscr{C}}}_{ij}(z)$. In particular, if the process is Markovian (more precisely, a continuous time Markov chain), then all the absolute inter-event times, $\{{T}_{i\to j},\,j\in A(i)\}$, for any event leaving state *i*, are exponentially distributed. Mathematically, ${T}_{i\to j}\sim Exp({\lambda }_{ij})$, as mentioned in Figs 1b and 2. If this is the case, it is well known that for any $i\in {\mathscr{S}}$ and $j\in A(i)$, we have

$${p}_{ij}={\mathbb{P}}(i\to j)=\frac{{\lambda }_{ij}}{{\sum }_{k\in A(i)}\,{\lambda }_{ik}},\,{{\mathscr{C}}}_{ij}(z)=E[{e}^{-z{T}_{i\to j}}|i\to j]=\frac{{\sum }_{k\in A(i)}\,{\lambda }_{ik}}{{\sum }_{k\in A(i)}\,{\lambda }_{ik}+z}.$$

If we substitute these expressions in Eq. (1), one obtains that the transforms ${ {\mathcal L} }_{i}(z)$ correspond to those of a phase-type distribution (for further details, see Proposition 1.2.2 in^13^), since ${T}_{i}({\rm{\Omega }})$ represents the absorption time for a continuous time Markov chain.

**Figure 3:**
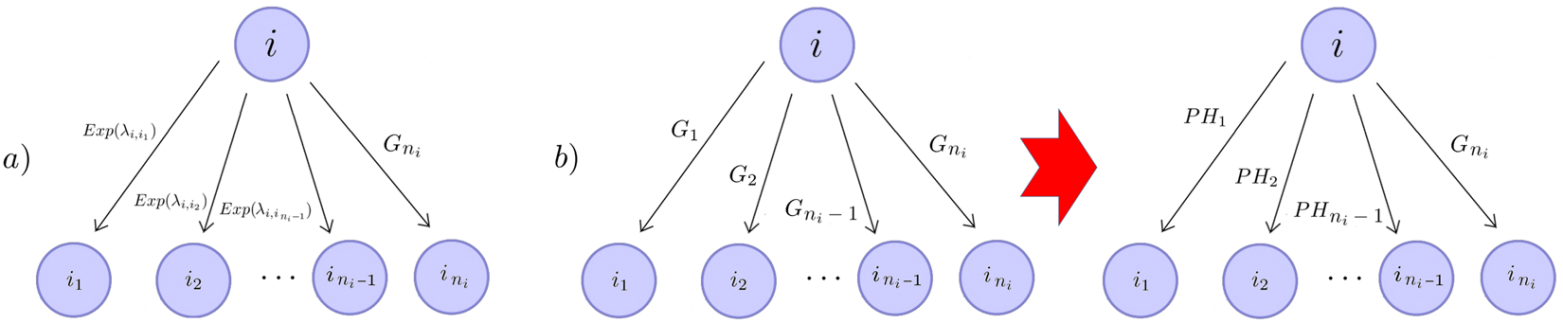
(**a**) From any given state $i\in {\mathscr{S}}$, the absolute waiting times to any state $j\in A(i)=\{{i}_{1},{i}_{2},\ldots ,{i}_{{n}_{i}-1}\}$ are exponentially distributed, ${T}_{i\to j}\sim Exp({\lambda }_{ij})$, except for at most one state ${i}_{{n}_{i}}$. (**b**) If the absolute waiting times are generally distributed, one can approximate all but one of these distributions by phase-type ones, and the methods described in this paper still apply.

When other distributions other than the exponential are considered, the expressions for the absolute waiting times are not known explicitly. Our aim is to consider alternative distributions to the exponential one for the absolute waiting times ${T}_{i\to j}$ with $i\in {\mathscr{S}}$, $j\in A(i)$, as represented in Fig. 3, so that we are still able to compute the probabilities ${p}_{ij}$ and the conditioned transforms ${{\mathscr{C}}}_{ij}(z)$, and to apply Eq. (1) and its related matrix analytic techniques^13^. In the next section we explore how more general distributions can be considered (under special circumstances), by making use of phase-type distributions. We illustrate our approach making use of several biological case studies in the following sections.

### Absolute waiting times, transition probabilities and conditioned waiting times for general distributions

Let us assume that for any state in our process, $i\in {\mathscr{S}}$, with $A(i)=\{{i}_{1},{i}_{2},\ldots ,{i}_{{n}_{i}}\}$, the absolute inter-event (or waiting) times ${T}_{i\to j}$ for $j\in \{{i}_{1},{i}_{2},\ldots ,{i}_{{n}_{i}-1}\}$, are exponentially distributed, that is, ${T}_{i\to j}\sim Exp({\lambda }_{ij})$, and that there is one state ${i}_{{n}_{i}}\in A(i)$ (the last one in the list *A*(*i*), without loss of generality) for which this time is generally distributed, ${T}_{i\to {i}_{{n}_{i}}}\sim {G}_{{n}_{i}}$ (see Fig. 3a). The distribution ${G}_{{n}_{i}}$ can be described in terms of its Laplace-Stieltjes transform, ${ {\mathcal L} }_{{G}_{{n}_{i}}}(z)=E[{e}^{-z{T}_{i\to {i}_{{n}_{i}}}}]$. Under these conditions, then it is possible to show (see Supplementary Information) that

2 $$\begin{array}{ccc}{p}_{i{i}_{{n}_{i}}} & = & {\mathbb{P}}(i\to {i}_{{n}_{i}})={{\mathscr{L}}}_{{G}_{{n}_{i}}}\,(\sum _{k={i}_{1}}^{{i}_{{n}_{i}-1}}\,{\lambda }_{ik}),\\ {p}_{ij} & = & {\mathbb{P}}(i\to j)=\frac{{\lambda }_{ij}}{\sum _{k={i}_{1}}^{{i}_{{n}_{i}-1}}\,{\lambda }_{ik}}\,[1-{{\mathscr{L}}}_{{G}_{{n}_{i}}}\,(\sum _{k={i}_{1}}^{{i}_{{n}_{i}-1}}\,{\lambda }_{ik})],\,j\in \{{i}_{1},{i}_{2},\ldots ,{i}_{{n}_{i}-1}\},\\ {{\mathscr{C}}}_{i{i}_{{n}_{i}}}(z) & = & \frac{{{\mathscr{L}}}_{{G}_{{n}_{i}}}\,(z+\sum _{k={i}_{1}}^{{i}_{{n}_{i}-1}}\,{\lambda }_{ik})}{{{\mathscr{L}}}_{{G}_{{n}_{i}}}\,(\sum _{k={i}_{1}}^{{i}_{{n}_{i}-1}}\,{\lambda }_{ik})},\\ {{\mathscr{C}}}_{ij}(z) & = & \frac{\sum _{k={i}_{1}}^{{i}_{{n}_{i}-1}}\,{\lambda }_{ik}}{\sum _{k={i}_{1}}^{{i}_{{n}_{i}-1}}\,{\lambda }_{ik}+z}\,\frac{[1-{{\mathscr{L}}}_{{G}_{{n}_{i}}}\,(z+\sum _{k={i}_{1}}^{{i}_{{n}_{i}-1}}\,{\lambda }_{ik})]}{[1-{{\mathscr{L}}}_{{G}_{{n}_{i}}}\,(\sum _{k={i}_{1}}^{{i}_{{n}_{i}-1}}\,{\lambda }_{ik})]},\,j\in \{{i}_{1},{i}_{2},\ldots ,{i}_{{n}_{i}-1}\}.\end{array}$$

Once these quantities are at hand, it is possible to apply Eq. (1) to compute the Laplace-Stieltjes transform of the first passage time ${T}_{i}({\rm{\Omega }})$, for an initial state $i\in {\mathscr{S}}$, to the given state of interest ${\rm{\Omega }}\in {\mathscr{S}}$.

In Fig. 3b we consider the more interesting case where given any initial state $i\in {\mathscr{S}}$, all events $i\to j$ leaving this state have absolute waiting times that are generally distributed, ${T}_{i\to {i}_{l}}\sim {G}_{l}$, $l\in \{1,2,\ldots ,{n}_{i}\}$. If this is the case, it is not possible to obtain closed form expressions of the kind given by Eq. (2). However, one can approximate all but one of these distributions (for instance, ${G}_{{n}_{i}}$) by ${n}_{i}-1$ phase-type distributions $P{H}_{j}$, $j\in \{1,\ldots ,{n}_{i}-1\}$, leading to the situation depicted in Fig. 3b *right*. Thus, by combining exact (general) distributions with arbitrarily accurate approximate phase-type distributions, we can capture the inherent nature of almost any process subject to the semi-Markov restriction. We refer the reader to Supplementary Information, where we have provided all the required mathematical details that allow us to obtain an analogous result to Eq. (2) for the scenario described in Fig. 3b. We note that the approximation of general distributions by phase-type ones is justified, since the family of phase-type (PH) distributions is dense in the family of non-negative continuous distributions (see Theorem 1.2.1 in^13^), and algorithmic approaches have been derived to identify these approximate PH distributions^13,16,17^. We illustrate this point in case study 2 (Figs 9 and 10, and Supplementary Information). Finally, since the expressions in Eq. (2) depend on the Laplace-Stieltjes transform ${ {\mathcal L} }_{{G}_{{n}_{i}}}(z)=E[{e}^{-z{T}_{i\to {i}_{{n}_{i}}}}]$, our methods can be implemented even when some of the general distributions in Fig. 3 are discrete or degenerate (*i*.*e*., deterministic) waiting time distributions (see Results).

## Results

In this section we illustrate our methods by considering three different biological scenarios: receptor-ligand interactions, viral replication and epidemiology. Our first objective is to illustrate the practical implementation of our theoretical methods. Our second objective is to show that the Markovian hypothesis, in which all waiting times are considered exponentially distributed, might not be a good approximation.

### Case study 1: kinetic proof-reading

**Figure 4:**
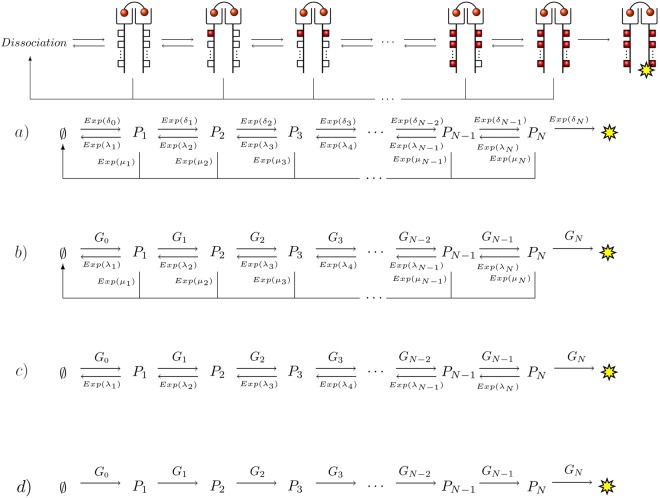
Kinetic proof-reading diagram. (**a**) Markovian model. (**b**) Model with generally distributed phosphorylation events. (**c** and **d**) Simplified models with some transitions removed. We are interested in studying the distribution of the time to reach the fully phosphorylated state.

Our first case study considers the signalling dynamics of membrane bound receptors after a ligand-receptor binding and phosphorylation event. In Fig. 4, we show the scenario in which intra-cellular signalling is triggered by a cross-linking event of a bivalent ligand to a receptor dimer^18^. This event is followed by a sequence of consecutive and reversible phosphorylation events, which ends when all the phosphorylation sites on the cytoplasmic tail of the receptor are active (or phosphorylated) and the receptor is internalised (thus, initiating the corresponding intra-cellular signalling cascade). If at any time the ligand dissociates from the receptor, the signalling is interrupted *catastrophically*^19^ and the process returns to the starting point (pre-binding). This process, known as kinetic proof-reading, provides a robust mechanism for ligand specificity and has been widely studied in the literature^1^, even from a stochastic (Markovian) perspective^20^.

In Fig. 4 we consider four possible scenarios for the process ${\mathscr{X}}$ that involves states $\{0,1,2,\ldots ,N\}\cup \{{\rm{\Omega }}\}$, where 0 represents the pre-binding state ($\varnothing$) and ${\rm{\Omega }}$ is the final (and fully) phosphorylated state, represented with a yellow star in Fig. 4. In Fig. 4a we summarise the Markovian view as introduced in ref.^20^. In this reference, all the transitions are Markovian and, thus, inter-event times are assumed to be exponentially distributed. In Fig. 4b we relax this assumption by incorporating generally distributed absolute waiting times for the phosphorylation events, so that the arguments of the Materials and Methods section still apply. In particular, we recognise that the event that allows the phosphorylation of a new site on the intra-cellular tail of the receptor requires (at least) the diffusion and binding to this tail of adaptor and scaffold proteins^21^, so that this waiting time could have a more complex form than exponential^22^. In Fig. 4c, we consider the case of a strong *agonist* (a ligand with a large affinity to the receptor, so that phosphorylation events occur in times short enough to assume that ligand dissociation cannot occur). Finally, in Fig. 4d, we assume that phosphorylation events are not reversible. This could represent a scenario where selected intra-cellular phosphatases have been deleted, so that dephosphorylation events cannot take place.

In our framework, these four scenarios can be summarised by providing the matrix ***A***(*z*) in Eq. (1). We recall that if an absolute waiting time is exponentially distributed, ${T}_{i\to j}\sim Exp({\lambda }_{ij})$, its Laplace-Stieltjes transform is given by

3 $${ {\mathcal L} }_{Exp({\lambda }_{ij})}\,(z)=E[{e}^{-z{T}_{i\to j}}]=\frac{{\lambda }_{ij}}{{\lambda }_{ij}+z}.$$

We denote by ${T}_{Exp({\lambda }_{j})}$, ${T}_{Exp({\mu }_{j})}$, ${T}_{Exp({\delta }_{j})}$ and ${T}_{{G}_{j}}$, the exponentially distributed and generally distributed absolute waiting times shown in Fig. 4. By providing suitable expressions for ${p}_{ij}$ and ${C}_{ij}(z)$ in Eq. (1), making use of the arguments in the Supplementary Information and Eq. (2), we can express Fig. 4 in matrix form. We note that, in all cases, the matrix structure captures the arrow structure of the diagrams shown in Fig. 4. Each of these matrices is given below.

$${{\bf{A}}}^{a)}\,(z)=(\begin{array}{ccccccc}1 & -\,{\textstyle \tfrac{{\delta }_{0}}{{\delta }_{0}+z}} & 0 & 0 & 0 & \ldots  & 0\\ -\,{\textstyle \tfrac{{\mu }_{1}+{\lambda }_{1}}{{\mu }_{1}+{\lambda }_{1}+{\delta }_{1}+z}} & 1 & -\,{\textstyle \tfrac{{\delta }_{1}}{{\mu }_{1}+{\lambda }_{1}+{\delta }_{1}+z}} & 0 & 0 & \ldots  & 0\\ -\,{\textstyle \tfrac{{\mu }_{2}}{{\mu }_{2}+{\lambda }_{2}+{\delta }_{2}+z}} & -\,{\textstyle \tfrac{{\lambda }_{2}}{{\mu }_{2}+{\lambda }_{2}+{\delta }_{2}+z}} & 1 & -\,{\textstyle \tfrac{{\delta }_{2}}{{\mu }_{2}+{\lambda }_{2}+{\delta }_{2}+z}} & 0 & \ldots  & 0\\ \ldots  & \ldots  & \ldots  & \ldots  & \ldots  & \ldots  & \ldots \\ -\,{\textstyle \tfrac{{\mu }_{N}}{{\mu }_{N}+{\lambda }_{N}+{\delta }_{N}+z}} & 0 & 0 & \ldots  & -\,{\textstyle \tfrac{{\lambda }_{N}}{{\mu }_{N}+{\lambda }_{N}+{\delta }_{N}+z}} & 1 & -\,{\textstyle \tfrac{{\delta }_{N}}{{\mu }_{N}+{\lambda }_{N}+{\delta }_{N}+z}}\\ 0 & 0 & 0 & \ldots  & 0 & 0 & 1\end{array})$$

$${{\bf{A}}}^{b)}(z)=(\begin{array}{ccccc}1 & -{{\mathscr{L}}}_{{G}_{0}}(z) & 0 & \ldots  & 0\\ -{\textstyle \tfrac{{\mu }_{1}+{\lambda }_{1}}{{\mu }_{1}+{\lambda }_{1}+z}}(1-{{\mathscr{L}}}_{{G}_{1}}({\mu }_{1}+{\lambda }_{1}+z)) & 1 & -{{\mathscr{L}}}_{{G}_{1}}({\mu }_{1}+{\lambda }_{1}+z) & \ldots  & 0\\ -{\textstyle \tfrac{{\mu }_{2}}{{\mu }_{2}+{\lambda }_{2}+z}}(1-{{\mathscr{L}}}_{{G}_{2}}({\mu }_{2}+{\lambda }_{2}+z)) & -{\textstyle \tfrac{{\lambda }_{2}}{{\mu }_{2}+{\lambda }_{2}+z}}(1-{{\mathscr{L}}}_{{G}_{2}}({\mu }_{2}+{\lambda }_{2}+z)) & 1 & \ldots  & 0\\ \ldots  & \ldots  & \ldots  & \ldots  & \ldots \\ -{\textstyle \tfrac{{\mu }_{N}}{{\mu }_{N}+{\lambda }_{N}+z}}(1-{{\mathscr{L}}}_{{G}_{N}}({\mu }_{N}+{\lambda }_{N}+z)) & 0 & \ldots  & 1 & -{{\mathscr{L}}}_{{G}_{N}}({\mu }_{N}+{\lambda }_{N}+z)\\ 0 & 0 & \ldots  & 0 & 1\end{array}).$$

We note that the case in Fig. 4a can be obtained from the latter matrix by setting the distributions ${G}_{j}\equiv Exp({\delta }_{j})$. In this case, ${ {\mathcal L} }_{{G}_{j}}(z)=\frac{{\delta }_{j}}{{\delta }_{j}+z}$ and the resulting **A**^*b*)^ (*z*) is exactly the matrix obtained for case *a*).

$${{\bf{A}}}^{c)}(z)=(\begin{array}{cccccc}1 & -{{\mathscr{L}}}_{{G}_{0}}(z) & 0 & 0 & \ldots  & 0\\ -{\textstyle \tfrac{{\lambda }_{1}}{{\lambda }_{1}+z}}(1-{{\mathscr{L}}}_{{G}_{1}}({\lambda }_{1}+z)) & 1 & -{{\mathscr{L}}}_{{G}_{1}}({\lambda }_{1}+z) & 0 & \ldots  & 0\\ 0 & -{\textstyle \tfrac{{\lambda }_{2}}{{\lambda }_{2}+z}}(1-{{\mathscr{L}}}_{{G}_{2}}({\lambda }_{2}+z)) & 1 & -{{\mathscr{L}}}_{{G}_{2}}({\lambda }_{2}+z) & \ldots  & 0\\ \ldots  & \ldots  & \ldots  & \ldots  & \ldots  & \ldots \\ 0 & 0 & \ldots  & -{\textstyle \tfrac{{\lambda }_{N}}{{\lambda }_{N}+z}}(1-{{\mathscr{L}}}_{{G}_{N}}({\lambda }_{N}+z)) & 1 & -{{\mathscr{L}}}_{{G}_{N}}({\lambda }_{N}+z)\\ 0 & 0 & \ldots  & 0 & 0 & 1\end{array}).$$

Again, this case can be seen as a particular case of *b*) when *μ*_*j*_ = 0 for $1\le j\le N$.

$${{\bf{A}}}^{d)}(z)=(\begin{array}{cccccc}1 & -{{\mathscr{L}}}_{{G}_{0}}(z) & 0 & 0 & \ldots  & 0\\ 0 & 1 & -{{\mathscr{L}}}_{{G}_{1}}(z) & 0 & \ldots  & 0\\ 0 & 0 & 1 & -{{\mathscr{L}}}_{{G}_{2}}(z) & \ldots  & 0\\ \ldots  & \ldots  & \ldots  & \ldots  & \ldots  & \ldots \\ 0 & 0 & \ldots  & 0 & 1 & -{{\mathscr{L}}}_{{G}_{N}}(z)\\ 0 & 0 & \ldots  & 0 & 0 & 1\end{array}).$$

As the latter matrix is bidiagonal, it is straightforward to see that in this case ${ {\mathcal L} }_{i}(z)=E[{e}^{-z{T}_{i}({\rm{\Omega }})}]=$ ${({{\bf{A}}}^{-1}(z))}_{i,N+1}={\prod }_{k=i}^{N}\,{ {\mathcal L} }_{{G}_{k}}(z)$, which is the well known result for a pure birth process^23^. That is, the time to reach state ${\rm{\Omega }}$, starting from state *i*, is the sum of the independent and generally distributed times ${T}_{i}({\rm{\Omega }})={T}_{{G}_{i}}+{T}_{{G}_{i+1}}+\cdots +{T}_{{G}_{N}}$, so that ${ {\mathcal L} }_{i}(z)$ is the product of their corresponding transforms. Moreover, if ${ {\mathcal L} }_{{G}_{k}}(z)=\lambda /(\lambda +z)$ for all *k* (that is, if ${G}_{k}\equiv Exp(\lambda )$ for some *λ* > 0), ${ {\mathcal L} }_{i}(z)$ is the Laplace-Stieltjes transform of an Erlang distribution, as one would expect.

The transforms ${ {\mathcal L} }_{i}(z)$ can be obtained by solving Eq. (1) for the matrices **A**(*z*) above. For the general case *b*), and if we define ${{\rm{\Delta }}}_{j}={\lambda }_{j}+{\mu }_{j}$, a recursive solution is given as follows:

$$\begin{array}{rcl}{h}_{0}(z) & = & 1.\\ {h}_{1}(z) & = & 1-\tfrac{{{\rm{\Delta }}}_{1}}{{{\rm{\Delta }}}_{1}+z}[1-{ {\mathcal L} }_{{G}_{1}}({{\rm{\Delta }}}_{1}+z)]{ {\mathcal L} }_{{G}_{0}}(z).\\ {h}_{2}(z) & = & 1-[1-{ {\mathcal L} }_{{G}_{2}}({{\rm{\Delta }}}_{2}+z)]{h}_{1}{(z)}^{-1}{ {\mathcal L} }_{{G}_{1}}({{\rm{\Delta }}}_{1}+z)(\tfrac{{\mu }_{2}{ {\mathcal L} }_{{G}_{0}}(z)+{\lambda }_{2}}{{{\rm{\Delta }}}_{2}+z}).\\ {h}_{3}(z) & = & 1-[1-{ {\mathcal L} }_{{G}_{3}}({{\rm{\Delta }}}_{3}+z)]{h}_{2}{(z)}^{-1}{ {\mathcal L} }_{{G}_{2}}({{\rm{\Delta }}}_{2}+z)(\tfrac{{\mu }_{3}{ {\mathcal L} }_{{G}_{0}}(z){h}_{1}{(z)}^{-1}{ {\mathcal L} }_{{G}_{1}}({{\rm{\Delta }}}_{1}+z)+{\lambda }_{3}}{{{\rm{\Delta }}}_{3}+z}).\end{array}$$

*For* $$k=4,\ldots ,N$$:

$$\begin{array}{rcl}{h}_{k}(z) & = & 1-[1-{ {\mathcal L} }_{{G}_{k}}({{\rm{\Delta }}}_{k}+z)]{h}_{k-1}{(z)}^{-1}{ {\mathcal L} }_{{G}_{k-1}}({{\rm{\Delta }}}_{k-1}\\  &  & +\,z)(\tfrac{{\mu }_{k}{ {\mathcal L} }_{{G}_{0}}(z){h}_{1}{(z)}^{-1}{ {\mathcal L} }_{{G}_{1}}({{\rm{\Delta }}}_{1}+z)\ldots {h}_{k-2}{(z)}^{-1}{ {\mathcal L} }_{{G}_{k-2}}({{\rm{\Delta }}}_{k-2}+z)+{\lambda }_{k}}{{{\rm{\Delta }}}_{k}+z}).\\ { {\mathcal L} }_{N}(z) & = & {h}_{N}{(z)}^{-1}{ {\mathcal L} }_{{G}_{N}}({{\rm{\Delta }}}_{N}+z).\end{array}$$

*For* $$k=N-1,N-2,\ldots ,0:$$

4 $${ {\mathcal L} }_{k}(z)={h}_{k}{(z)}^{-1}{ {\mathcal L} }_{{G}_{k}}({{\rm{\Delta }}}_{k}+z){ {\mathcal L} }_{k+1}(z).$$

This recursive scheme is also valid for *a*) and *c*), since these are particular cases of case *b*). We note that Eq. (4) for the scenario considered in Fig. 4d would be given by, for any initial state $0\le i\le N$, the following expression

$${ {\mathcal L} }_{i}(z)=\prod _{k=i}^{N}\,{ {\mathcal L} }_{{G}_{k}}(z)\Rightarrow E[{T}_{0}({\rm{\Omega }})]=\sum _{k=0}^{N}\,E[{T}_{k\to k+1}].$$

**Figure 5:**
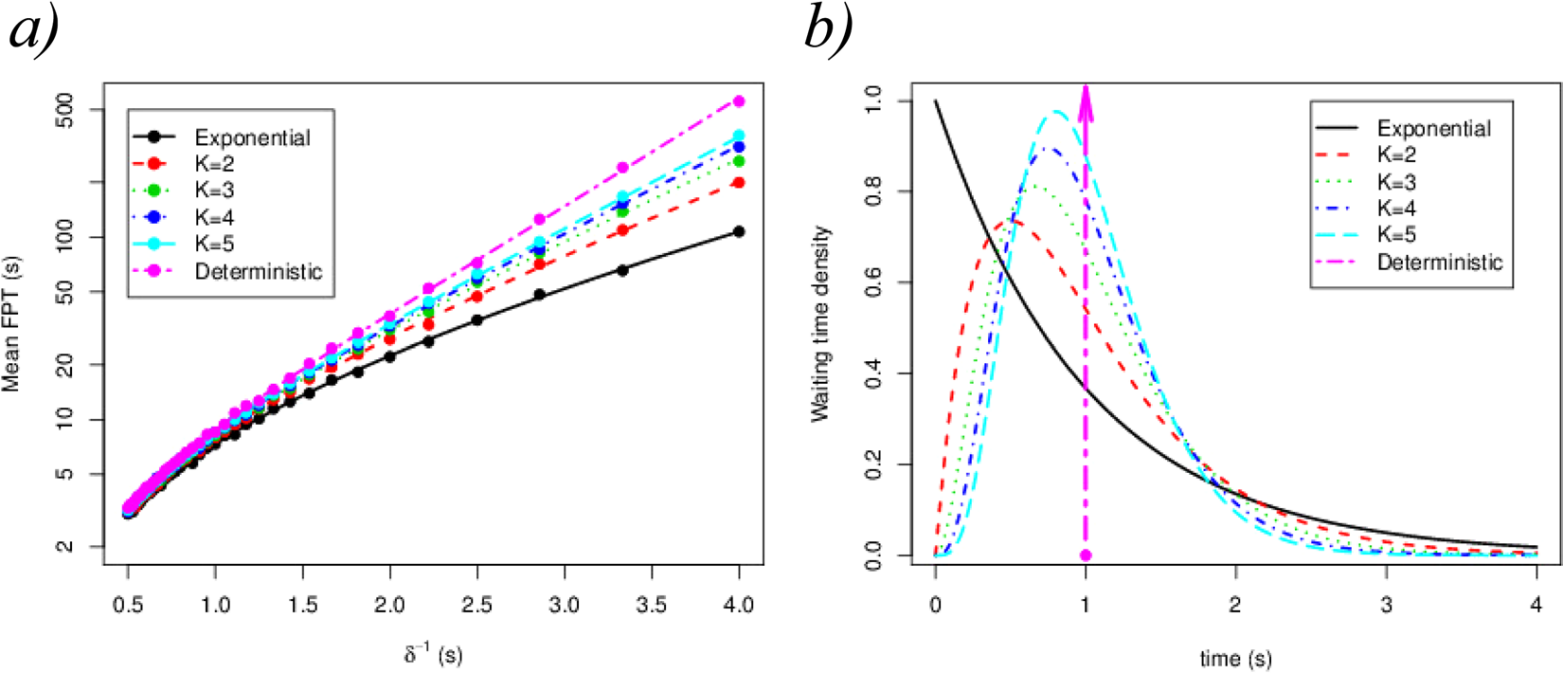
(**a**) Mean FPT $E[{T}_{0}({\rm{\Omega }})]$ from pre-binding (state 0) until signalling (state ${\rm{\Omega }}$) for the model described in Fig. 4b. The probability distributions considered for the absolute waiting time are shown in panel (b). Every waiting time distribution, *G*, in panel (b) has the same mean, ${\delta }^{-1}=1s$, but different shape. The value *K* refers to the shape parameter of the gamma distribution ($K=1$ corresponds to the exponential distribution). We have considered $N=4$, ${\lambda }_{k}=0.2{s}^{-1}$ and ${\mu }_{k}=0.1{s}^{-1}$ for all *k* in Fig. 4, for illustrative purposes.

Namely, the mean time to reach state ${\rm{\Omega }}$ (mean first passage time (FPT)) starting at 0, $E[{T}_{0}({\rm{\Omega }})]$, is the sum of the mean times spent in every transition of the process. This result is also what one would expect under the assumption that every transition can be described by an exponential rate *δ*_*k*_, where we obtain

$$E[{T}_{0}({\rm{\Omega }})]=\sum _{k=0}^{N}\,\frac{1}{{\delta }_{k}},$$

which only depends on the mean times of each transition, ${\delta }_{k}^{-1}$. However, the mean of each transition event is not enough when analysing the mean FPT to state ${\rm{\Omega }}$ if general distributions are considered for scenarios more complex than Fig. 4d, such as those described by Fig. 4b,c. In particular, when studying the mean FPT to reach state ${\rm{\Omega }}$ from the initial state 0 (see Fig. 5), we set the mean time for a phosphorylation event to be the same, $E[{T}_{{G}_{k}}]={\delta }^{-1}$ independently of *k* and we set the distribution *G* (${G}_{k}\equiv G$ for all *k*) and consider the case given by Fig. 4a and that of Fig. 4b (that is, $G\equiv Exp(\delta )$ or other general distributions). In particular, we use an exponential distribution (corresponding to Fig. 4a) and gamma distributions with shape $K=2,\ldots ,5$ and a deterministic distribution with time *δ*^−1^ (see densities in Fig. 5b). The symbols in Fig. 5a stand for the numerical simulations of the diagram in Fig. 4 and the lines for the theoretical prediction provided by Eq. (4).

Figure 5 clearly shows that the shape of the distribution *G* can dramatically change the estimated mean signalling time $E[{T}_{0}({\rm{\Omega }})]$ (note that the vertical axis is a logarithmic scale), even when $E[{T}_{G}]={\delta }^{-1}$ is the same. We note that an important result derived from our approach, and reported in the Supplementary Information, is that from the empirical absolute waiting time distributions, we can relate the conditioned times, ${T}_{i\to j}|i\to j$ to the absolute ones, ${T}_{i\to j}$. Finally, we stress the fact that these general results can be applied to the generic case (Fig. 4b), or in the presence of an agonist ligand (Fig. 4c) or in the absence of phosphatases (Fig. 4d).

### Case study 2: virion production

**Figure 6:**
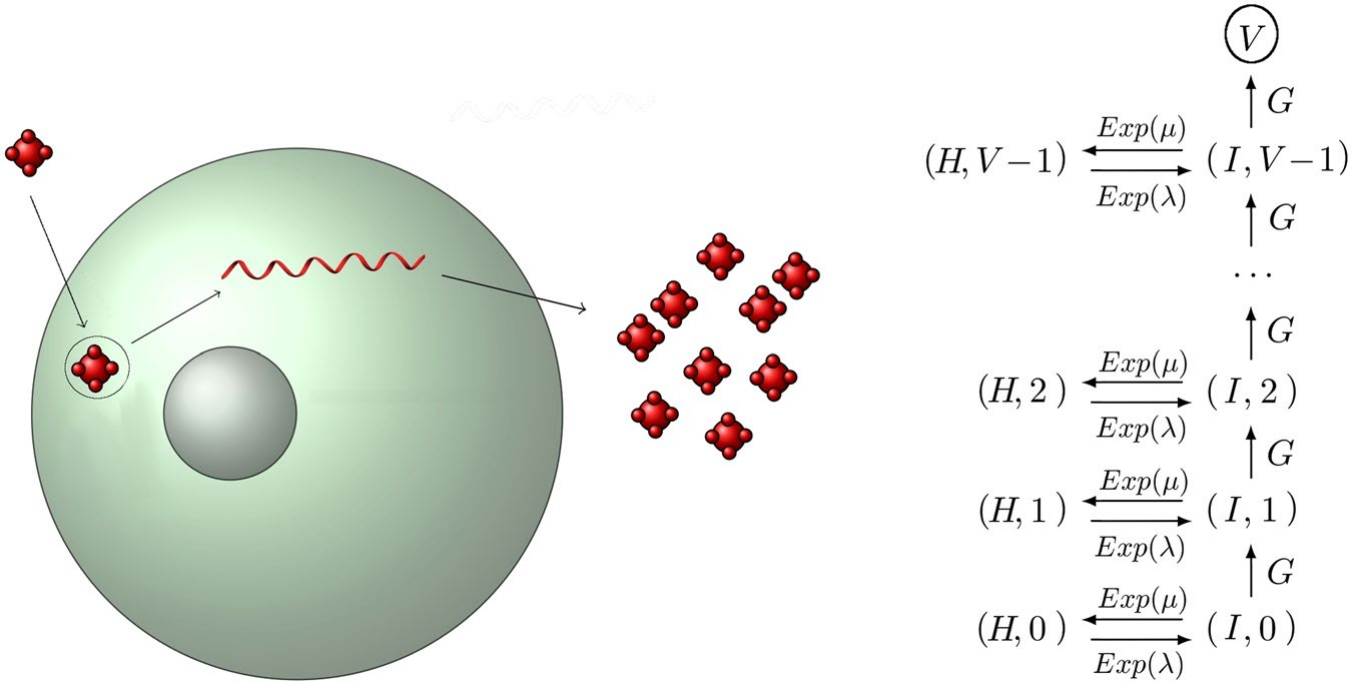
Viral budding for a cell that switches between healthy (*H*) and infected (*I*) states.

Our next case study involves the continuous release of virions from infected cells. Our model consists of a cell that can be in two states: *healthy* (*H*) or *infected* (*I*). In the latter, the cell releases one virion at a time after generally distributed times, *T*_*G*_, with common general distribution *G*, and Laplace-Stieltjes transform ${ {\mathcal L} }_{G}(z)=E[{e}^{-z{T}_{G}}]$. The transition between the infected and healthy states is assumed to be exponentially distributed (with rates *λ* and *μ*), as shown in Fig. 6. A natural question is to obtain the distribution of the total time, ${T}_{(H,0)}(V)$, it takes to produce *V* virions, starting from state (*H*, 0), that is, at time *t* = 0 the cell is in the healthy state *H* and no virions have been released yet.

Using the definitions provided in the Materials and Methods section, and by ordering the space of states as follows

$$(H,\,0)\,\prec \,(I,\,0)\,\prec \,(H,\,1)\,\prec \,(I,\,1)\,\prec \,\cdots \,\prec \,(H,\,V-1)\,\prec \,(I,\,V-1)\,\prec \,V,$$

we can write the matrix

$${\bf{A}}(z)=(\begin{array}{ccccccc}1 & -\,{\textstyle \tfrac{\lambda }{\lambda +z}} & 0 & 0 & 0 & \ldots  & 0\\ -\,{\textstyle \tfrac{\mu }{\mu +z}}(1-{{\mathscr{L}}}_{G}(\mu +z)) & 1 & 0 & -{{\mathscr{L}}}_{G}(\mu +z) & 0 & \ldots  & 0\\ 0 & 0 & 1 & -\,{\textstyle \tfrac{\lambda }{\lambda +z}} & 0 & \ldots  & 0\\ 0 & 0 & -\,{\textstyle \tfrac{\mu }{\mu +z}}(1-{{\mathscr{L}}}_{G}(\mu +z)) & 1 & 0 & \ldots  & 0\\ \ldots  & \ldots  & \ldots  & \ldots  & \ldots  & \ldots  & \ldots \\ 0 & 0 & \ldots  & \ldots  & \ldots  & 1 & -{{\mathscr{L}}}_{G}(\mu +z)\end{array}).$$

Following the same procedure as in the previous case study, we find that for the initial state (*H*, 0),

$${ {\mathcal L} }_{(H,0)}\,(z)=E[{e}^{-z{T}_{(H,0)}(V)}]=\tfrac{\lambda }{\lambda +z}{(\tfrac{(\lambda +z)(\mu +z){ {\mathcal L} }_{G}(\mu +z)}{(\lambda \mu { {\mathcal L} }_{G}(\mu +z)+z(\lambda +\mu +z))})}^{V},$$

and thus, the mean time to produce *V* virions is given by

5 $$E[{T}_{(H,0)}(V)]=-\,{\tfrac{d{ {\mathcal L} }_{(H,0)}(z)}{dz}|}_{z=0}=\tfrac{{ {\mathcal L} }_{G}(\mu )\,(\mu -V(\lambda +\mu ))+V(\lambda +\mu )}{\lambda \mu { {\mathcal L} }_{G}(\mu )}=\frac{1}{\lambda }+V\,(\frac{1}{\mu }+\frac{1}{\lambda })\,[{ {\mathcal L} }_{G}{(\mu )}^{-1}-1].$$

This expression can be easily understood: the mean time to produce *V* virions can be split as the sum of the mean time to get the cell infected (1/*λ*) and the mean time per new virion production, ${\rm{\Delta }}T=(\frac{1}{\mu }+\frac{1}{\lambda })\,[{ {\mathcal L} }_{G}{(\mu )}^{-1}-\mathrm{1]}$, times the number of required virions, *V*. We note that ${\rm{\Delta }}T$ depends on ${ {\mathcal L} }_{G}(\mu )$. Thus, the shape of the general waiting time distribution *G* changes the prediction of the mean time to produce *V* virions. For a purely Markovian process, $G\equiv Exp(\frac{1}{\tau })$, where $\tau$ denotes the mean virion production time, we have

6 $${ {\mathcal L} }_{G}{(\mu )}^{-1}-1=\mu \tau ,$$

which only depends on $E[{T}_{G}]=\tau$. However, for more general distributions, ${ {\mathcal L} }_{G}(\mu )$ would depend also on all the moments (or cumulants, such as the variance, *σ*^2^) of the general distribution *G*, since

$${ {\mathcal L} }_{G}{(\mu )}^{-1}-1=\mu \tau +\frac{1}{2}{\mu }^{2}({\tau }^{2}-{\sigma }^{2})+\ldots ({\rm{higher}}\,{\rm{moments}}).$$

These differences are highlighted in the numerical simulations which follow.

**Figure 7:**
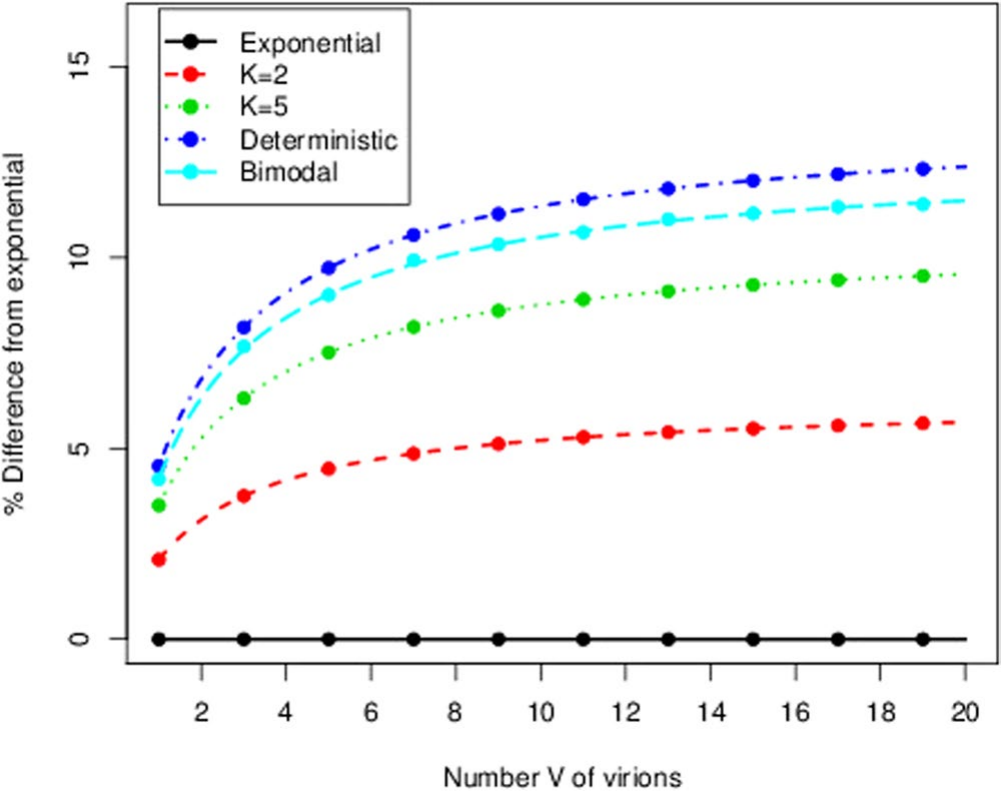
Percentage difference between the mean FPT $E[{T}_{(H,0)}(V)]$ under the Markovian hypothesis and under general distributions *G* for each budding event (see Fig. 6). A common mean $\tau =0.5\,h$ has been chosen, with $\mu =\lambda =0.5\,{h}^{-1}$. The label *bimodal* refers to a mixture $qN({\tau }_{1},{\sigma }^{2})+(1-q)N({\tau }_{2},{\sigma }^{2})$ of Gaussian distributions with values $\sigma =0.05$, $q=0.26$, ${\tau }_{1}=0.26\,h$ and ${\tau }_{2}=0.58\,h$, so that $\tau =q{\tau }_{1}+(1-q){\tau }_{2}=0.5\,h$. Dots show the results from stochastic simulations and lines show the theoretical results obtained from our matrix analytic approach.

As we discussed above, the time to produce *V* virions is the sum of the waiting time until the first infection plus *V* times the time in a production-recovery-reinfection cycle. In Fig. 7 we show the percentage difference between the mean FPT $E[{T}_{(H,0)}(V)]$ under the Markovian hypothesis ($G\equiv Exp(\tfrac{1}{\tau })$ in Fig. 6) and under generally distributed absolute waiting times for the budding event. The bimodal distribution is a mixture of two Gaussian distributions centered at times ${\tau }_{1}$ and ${\tau }_{2}$, inspired by the experimental observations discussed in ref.^4^. We have chosen the parameters of the general distributions so that they have the same mean, $\tau$, as the exponential case.

Inspection of Fig. 7 reveals two important features. First, the numerical simulations follow the theoretical predictions for any choice of distribution *G*. Secondly, and as we have already discussed in the Results section, the shape of the distribution *G*, and not only its mean, is an important factor to estimate the mean time to produce *V* virions. For the parameters used in Fig. 7, these differences can be as large as 12%. In fact, these differences will be larger or smaller depending on the form of the Laplace-Stieljes transform of the absolute waiting time distribution considered, and whether this form can or cannot be suitably captured by its first few moments (for instance, if the distribution is not monotonic). The largest differences are obtained when $G\equiv Deterministic\equiv \tau$ is considered. This fact, which would not be true in general for an arbitrary model, is true for the particular model described in Fig. 6. Note that, from Jensen’s inequality, for any arbitrary density function of a random variable *X*, the following inequality is satisfied

$$\exp \,(\,-\,zE[X])\le E[\exp (\,-\,zX)].$$

If the term on the right is the definition of the Laplace transform of the general distribution *G*, and considering that the general distribution has mean $\tau$, we find

$${e}^{-z\tau }\le { {\mathcal L} }_{G}(z)$$

Interestingly, ${e}^{-z\tau }$ is the Laplace transform of the deterministic distribution. Hence, as the mean time to produce *V* virions is proportional to ${ {\mathcal L} }_{G}{(\mu )}^{-1}-1$, by Eq. (5), this proves that the deterministic distribution constitutes an upper bound for $E[{T}_{(H,0)}(V)]$ in Fig. 7.

**Figure 8:**
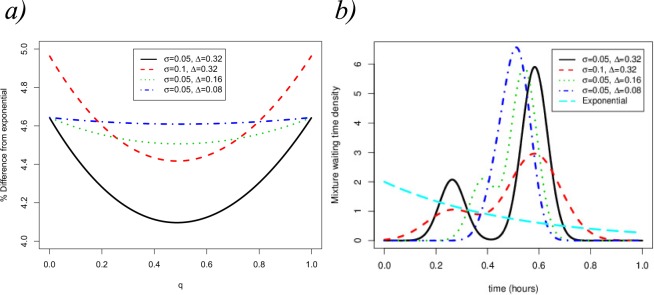
(**a**) Relative difference in the mean time to produce *V* = 1 virions between the Markovian hypothesis $(G\equiv Exp(\frac{1}{\tau }))$ and the choice of *G* as a mixture of Gaussian distributions with different *shifts*, ${\rm{\Delta }}$, and variances, *σ*^2^. We note that values of ${\tau }_{1}$ and ${\tau }_{2}$ are adjusted according to Eq. (7), so that $E[{T}_{G}]=\tau$. (**b**) Plot of the distribution *G* for *q* = 0.26.

We further illustrate the impact of the particular choice of the distribution *G* in the first passage time under analysis by plotting (Fig. 8) the theoretical relative difference between the Markovian hypothesis $(G\equiv Exp(\tfrac{1}{\tau }))$ and the choice of *G* as a mixture of Gaussian distributions of the form

$${T}_{G}\sim qN\,({\tau }_{1},{\sigma }^{2})+(1-q)N\,({\tau }_{2},{\sigma }^{2})$$

where $\tau$ is the mean time of the exponential distribution as discussed in Eq. (6) and we vary, keeping $\tau$ fixed, as follows

7 $${\tau }_{2}-{\tau }_{1}={\rm{\Delta }},\,q{\tau }_{1}+(1-q){\tau }_{2}=\tau .$$

The parameter *σ* accounts for the common dispersion around each peak of the bimodal distribution and ${\rm{\Delta }}$ for the shift between the peaks. From Fig. 8 we can see that both parameters ${\rm{\Delta }}$ and *σ* have an impact on the % difference. Furthermore, Fig. 8 suggests that a minimal difference is obtained for *q* = 0.5, which represents the mixture of two Gaussian distributions with equal probability. It is worth noting that using an exponential distribution always underestimates the mean FPT.

**Figure 9:**
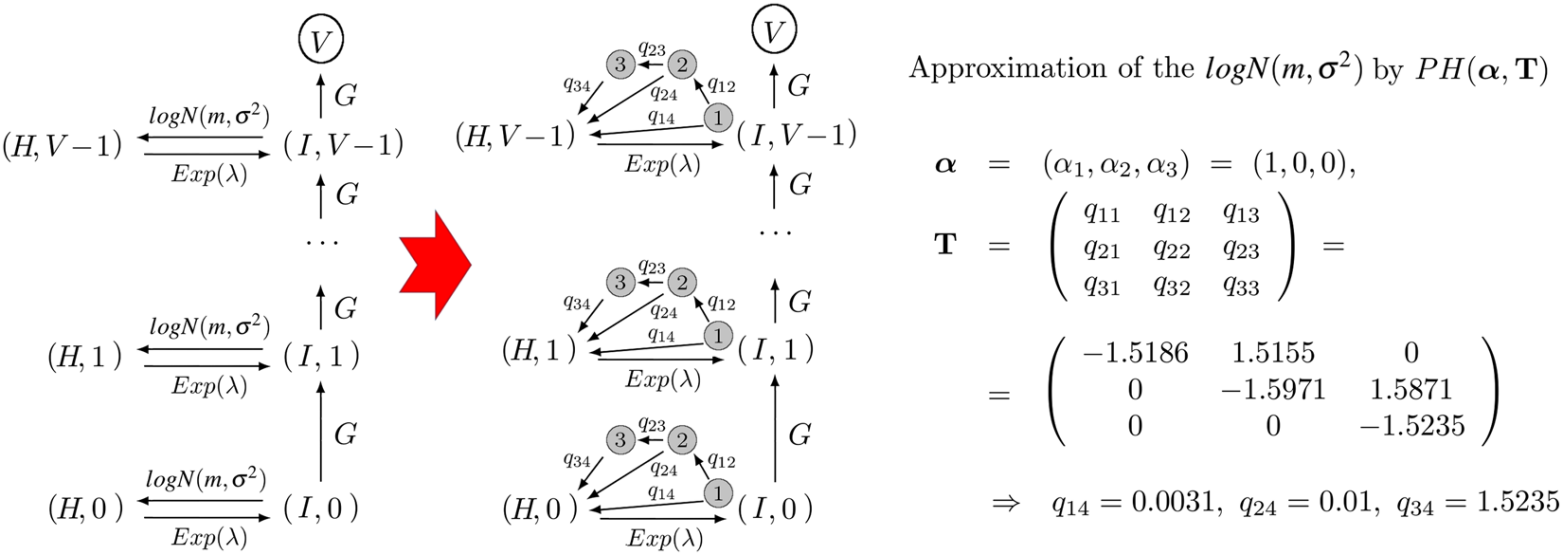
Process in Fig. 6 where recovery of the cell occurs after a *log*$N(m,\,{\sigma }^{2})$ distributed time, instead of *Exp*(*μ*). We consider *m* = 0.57 h, *σ* = 0.5 h so that 1/*μ* = 2 h. We approximate the log-normal recovery time with a phase-type distribution, *PH*(***α***, **T**), using three phases.

An additional strength of our approach is that we can extend these results to the case of phase-type (PH) waiting times. In particular, we can study a general scenario containing, for some initial state *i*, *n*_*i*_ general waiting time distributions, by approximating the process as follows: one such waiting time would be generally distributed and the remaining *n*_*i*_ − 1 waiting times would be PH distributions, with these PH distributions approximating the *n*_*i*_ − 1 general ones with a desired degree of accuracy (see Fig. 3). This is justified by the fact that any non-negative continuous distribution can be approximated by a PH distribution^13^, [Theorem 1.2.1]. To test the accuracy of this approximation within our framework, we have simulated this case study, but replacing the recovery event (originally an exponential distribution with rate *μ*) by a log-normal distribution with the same mean *μ*^−1^ and standard deviation *σ* = 0.5. We note that the use of a phase-type distribution as an approximation is equivalent to adding a number of ghost states in the system, see Fig. 9.

**Figure 10:**
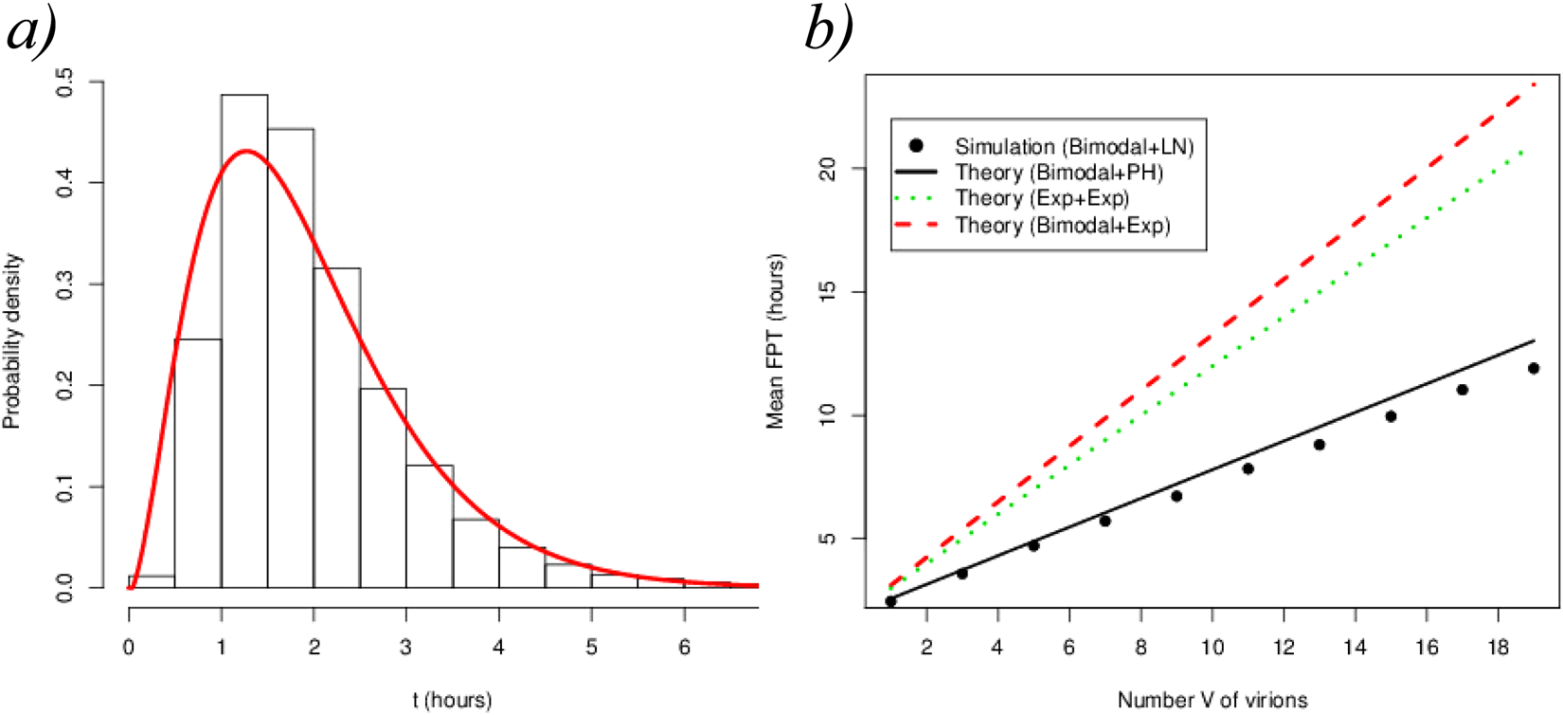
(**a**) Bars: histogram of the log-normal distribution *log*$N(m,\,{\sigma }^{2})$ with *m* = 0.57 h, *σ* = 0.5 h so that 1/*μ* = 2 h. Red solid line: probability density of the three-dimensional *PH*(***α***, **T**) approximation of the log-normal distribution. (**b**) Black circles: simulation of case study 2 with a mixture of Gaussian distributions for budding events and a log-normal one for the recovery events. The black solid line represents the theoretical prediction when replacing the log-normal by a PH distribution in panel (a). The red dashed line represents the theoretical prediction using a mixture of Gaussian distributions for the budding event and an exponential one for the recovery transition. The green dotted line represents the complete Markovian hypothesis.

Figure 10a shows a (black) histogram obtained by drawing 10^4^ sampled values from the log-normal distribution against the density curve (red) of the phase-type distribution *PH*(*α*, **T**) defined by

$$\begin{array}{rcl}\alpha  & = & (1,0,0),\\ {\bf{T}} & = & (\begin{array}{ccc}-1.5186 & 1.5155 & 0\\ 0 & -1.5971 & 1.5871\\ 0 & 0 & -1.5235\end{array}),\end{array}$$

which has been obtained using the PhaseType R package^16^. Figure 10a allows us to compare a three-dimensional PH (approximation) probability density (solid red line) to a histogram (black) of the corresponding log-normal distribution. The mean time, obtained by stochastic simulations, to produce *V* virions combining a mixture distribution for *G*, as defined above (budding events), and a log-normal one for recovery events (instead of *Exp*(*μ*)) is given by the filled black circles in Fig. 10b. Different approximations, where these two distributions are replaced either by an exponential one or by a PH distribution, yield the theoretically computed curves in Fig. 10b. It is to be expected that higher dimensional PH approximations of the log-normal would result in better approximations. Nevertheless, as shown in Fig. 10b, the black solid line is the theoretical prediction arising when the log-normal recovery time distribution is substituted by this PH distribution (see Supplementary Information for further details), while keeping the general bimodal distribution *G* for the budding events. The prediction of the theory captures accurately the simulations and shows the large overestimations arising from different approximations that make use of exponentially distributed waiting times. We note that only the bimodal plus PH theoretical approximation can account for the simulation results that correspond to the bimodal plus log-normal *real* system.

### Case study 3: interventions in epidemiology

Our last case deals with an epidemiological problem. We consider a medical doctor working in a hospital ward in which some bacteria have been detected to be spreading among patients and healthcare workers (HCWs). When this outbreak is detected (*t* = 0), this doctor is in a healthy (*susceptible*) state, and she/he can become infected due to treating the (potentially infected) patients at the ward during this outbreak. We consider the following assumptions:

(i) The outbreak finishes in an average time $\frac{1}{\delta }$, which we consider to be exponentially distributed, *Exp*(*δ*).
(ii) During this outbreak, the doctor can get infected after some average time $\frac{1}{\beta }$, which we consider to be exponentially distributed, *Exp*(*β*).
(iii) Once the doctor is infected, she/he can naturally recover after a mean time $\frac{1}{\gamma }$, and we consider this time to be exponentially distributed, *Exp*(*γ*).
  The Markovian hypotheses above are the usual ones when studying this type of processes from a stochastic perspective. However, we also incorporate generally distributed times to describe the following hospital surveillance (*i*.*e*., screening) policies:
(iv) Since the detection of the outbreak (*t* = 0), a new policy has been implemented so that every HCW is screened, every *B* days, to check if she/he is infected. If the HCW is found to be infected, she/he is sent off-duty.
(v) Once the HCW is sent off-duty, she/he is given some treatment so that her/his recovery occurs after a time that is exponentially distributed, $Exp(\tilde{\gamma })$, with $\tilde{\gamma }\ge \gamma$, and she/he is screened every *M* days to check for a complete recovery.

**Figure 11:**
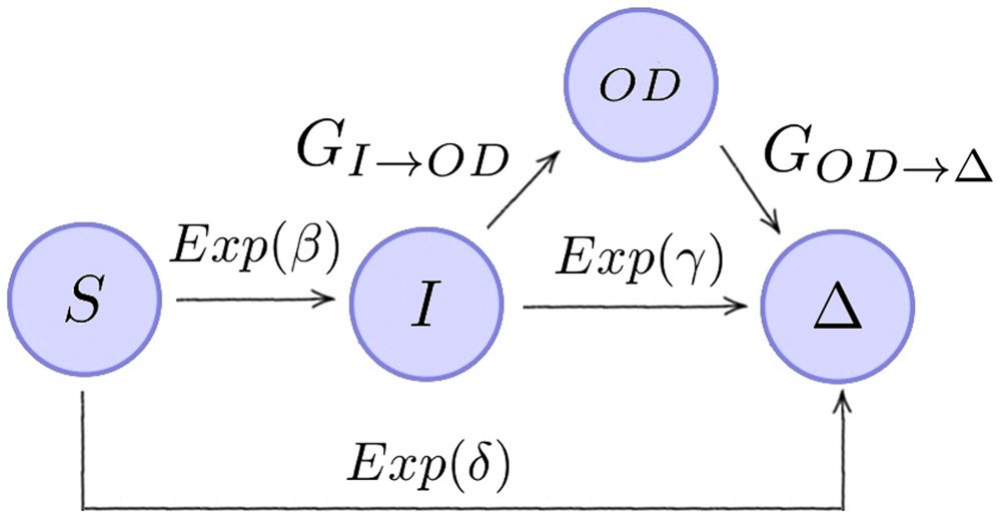
Single HCW epidemic model. General distributions ${G}_{I\to OD}$ and ${G}_{OD\to {\rm{\Delta }}}$ correspond to screening policies (iv) and (v) described in the main text.

We are interested in studying the process until the outbreak ends (if the HCW under surveillance does not become infected), or until the recovery of this individual is completed (if she/he suffers the infection). In order to analyse this process, we consider the diagram in Fig. 11. We study the process until it reaches the state ${\rm{\Delta }}$, which represents either the end of the outbreak at the hospital ward without the HCW under study becoming infected, or the recovery of this HCW if she/he suffers the infection.

The general distribution ${G}_{I\to OD}$ describes the time ${T}_{I\to OD}$ that it takes to detect the infection of the HCW, by means of the screening policy (SP) implemented in the hospital ward, defined in (iv). Thus, once the HCW is infected, the time ${T}_{I\to OD}$ for the infection of this HCW to be detected depends on how close to the next screening event this infection event actually occurred. We note that the time to infection of the individual, conditioned on this infection occurring, ${T}_{S\to I}|S\to I\sim Exp(\beta +\delta )$. This is true from the properties of the exponential distribution, thus the distribution of ${T}_{I\to OD}$ can be described in terms of its density function as follows

$$\begin{array}{rcl}{f}_{{T}_{I\to OD}}(t) & = & \sum _{k=0}^{+\infty }\,{f}_{{T}_{S\to I}|S\to I}((k+1)B-t)=\sum _{k=0}^{+\infty }\,(\beta +\delta ){e}^{-(\beta +\delta )((k+1)B-t)}\\  & = & (\beta +\delta ){e}^{(\beta +\delta )(t-B)}\,\sum _{k=0}^{+\infty }\,{e}^{-(\beta +\delta )kB}=\frac{(\beta +\delta ){e}^{(\beta +\delta )(t-B)}}{1-{e}^{-(\beta +\delta )B}},\,0 < t < B.\end{array}$$

Thus, the Laplace-Stieltjes transform of the time ${T}_{I\to OD}\sim {G}_{I\to OD}$ is given by

$${ {\mathcal L} }_{{G}_{I\to OD}}(z)={\int }_{0}^{B}\,{e}^{-zt}\,dt\frac{(\beta +\delta ){e}^{(\beta +\delta )(t-B)}}{1-{e}^{-(\beta +\delta )B}}=\frac{(\beta +\delta ){e}^{-(\beta +\delta )B}[{e}^{(\beta +\delta -z)B}-1]}{(\beta +\delta -z)\,[1-{e}^{-(\beta +\delta )B}]}.$$

On the other hand, the general distribution ${G}_{OD\to {\rm{\Delta }}}$ describes the time ${T}_{OD\to {\rm{\Delta }}}$ that it takes to detect the recovery of the off-duty HCW. That is, the sum of the times for the individual to recover and then for this recovery to be detected by the screening policy in (v). From the SP (v), it is clear that ${T}_{OD\to {\rm{\Delta }}}$ is a discrete random variable with the following probability mass function

$${\mathbb{P}}({T}_{OD\to {\rm{\Delta }}}=kM)={\mathbb{P}}((k-1)M < Exp(\tilde{\gamma }) < kM)={e}^{-\tilde{\gamma }(k-1)M}-{e}^{-\tilde{\gamma }kM},\,k\in \{1,2,3,\ldots \}.$$

Hence, the Laplace-Stieltjes transform of this distribution is given by

$${ {\mathcal L} }_{{G}_{OD\to {\rm{\Delta }}}}(z)=\sum _{k=1}^{+\infty }\,{e}^{-zkM}({e}^{-\tilde{\gamma }(k-1)M}-{e}^{-\tilde{\gamma }kM})=\frac{{e}^{-(z+\tilde{\gamma })M}({e}^{\tilde{\gamma }M}-1)}{1-{e}^{-(z+\tilde{\gamma })M}}.$$

We now order the states as follows: $S\,\prec \,I\,\prec \,OD\,\prec \,{\rm{\Delta }}$, so that the matrix **A**(*z*) is given by

8 $${\bf{A}}(z)=(\begin{array}{cccc}1 & -\,\frac{\beta }{\beta +\delta +z} & 0 & -\,\frac{\delta }{\beta +\delta +z}\\ 0 & 1 & -{ {\mathcal L} }_{{G}_{I\to OD}}(z+\gamma ) & -\,\frac{\gamma }{\gamma +z}(1-{ {\mathcal L} }_{{G}_{I\to OD}}(\gamma +z))\\ 0 & 0 & 1 & -{ {\mathcal L} }_{{G}_{OD\to {\rm{\Delta }}}}(z)\\ 0 & 0 & 0 & 1\end{array}).$$

We conclude then, that the Laplace-Stieltjes transform of the time ${T}_{S}({\rm{\Delta }})$ to reach state ${\rm{\Delta }}$, from the initial state *S*, ${ {\mathcal L} }_{S}(z)=E[{e}^{-z{T}_{S}({\rm{\Delta }})}]$, is given by

9 $${ {\mathcal L} }_{S}(z)=\tfrac{\beta (\beta +\delta )\,({e}^{\hat{\gamma }M}-1)\,({e}^{B(\beta -\gamma +\delta -z)}-1){e}^{B(-\beta -\delta )+M(-\hat{\gamma }-z)}}{(1-{e}^{B(-\beta -\delta )})\,(1-{e}^{M(-\hat{\gamma }-z)})\,(\beta +\delta +z)\,(\beta -\gamma +\delta -z)}+\tfrac{\beta \gamma (1-\tfrac{(\beta +\delta ){e}^{B(-\beta -\delta )}({e}^{B(\beta -\gamma +\delta -z)}-1)}{(1-{e}^{B(-\beta -\delta )})\,(\beta -\gamma +\delta -z)})}{(\gamma +z)\,(\beta +\delta +z)}+\tfrac{\delta }{\beta +\delta +z},$$

and the mean of the distribution is given by

10 $$\begin{array}{rcl}E[{T}_{S}] & = & \tfrac{1}{\gamma (\beta +\delta )\,[{e}^{\hat{\gamma }M}-1)\,(\beta -\gamma +\delta )\,({e}^{B(\beta +\delta )}-1)}{e}^{-B\gamma }(\,-\,(\beta +\gamma )\,(\beta -\gamma \\  &  & +\,\delta ){e}^{B(\beta +\gamma +\delta )}+\beta (\beta +\delta ){e}^{B(\beta +\delta )}\\  &  & -\,\gamma {e}^{B\gamma }(\gamma -\delta )+\beta (\beta +\delta )\,(\gamma M-1){e}^{B(\beta +\delta )+\hat{\gamma }M}\\  &  & +\,(\beta +\gamma )\,(\beta -\gamma +\delta ){e}^{B(\beta +\gamma +\delta )+\hat{\gamma }M}\\  &  & +\,\gamma {e}^{B\gamma +\hat{\gamma }M}(\gamma -\delta -\beta M(\beta +\delta ))].\end{array}$$

In order to show how this approach can also be applied to compute other quantities of interest, and not only first passage times, we now calculate the following probabilities: the probability of the medical doctor not becoming infected, ${p}_{S}(\overline{INF})$, suffering the infection and being sent off-duty, ${p}_{S}(INF\cap DET)$, and suffering the infection but not being detected ${p}_{S}(INF\cap \overline{DET})$. We know that

$${p}_{S}(\overline{INF})+{p}_{S}(INF\cap DET)+{p}_{S}(INF\cap \overline{DET})=1,$$

with

$$\begin{array}{rcl}{p}_{S}(\overline{INF}) & = & \frac{\delta }{\beta +\delta },\\ {p}_{S}(INF) & = & {p}_{S}(INF\cap DET)+{p}_{S}(INF\cap \overline{DET})=\frac{\beta }{\beta +\delta }.\end{array}$$

To compute the probabilities of being infected and detected, or infected but not detected, we can use the following system of equations obtained from a first step argument

$$\begin{array}{rcl}{p}_{S}(INF\cap DET) & = & {p}_{SI}{p}_{I}(INF\cap DET)+{p}_{S{\rm{\Delta }}}{p}_{{\rm{\Delta }}}(INF\cap DET),\\ {p}_{I}(INF\cap DET) & = & {p}_{I,OD}{p}_{OD}(INF\cap DET)+{p}_{I{\rm{\Delta }}}{p}_{{\rm{\Delta }}}(INF\cap DET),\end{array}$$

where ${p}_{ij}={\mathbb{P}}(i\to j)$ can be obtained from the arguments described in the Materials and Methods section and the Supplementary Information, and boundary conditions ${p}_{{\rm{\Delta }}}(INF\cap DET)=0$, ${p}_{OD}(INF\cap DET)=1$. Thus, we get

$$\begin{array}{rcl}{p}_{S}(INF\cap DET) & = & {p}_{SI}{p}_{I}(INF\cap DET)={\mathbb{P}}(S\to I){\mathbb{P}}(I\to OD)\\  & = & \tfrac{\beta }{\beta +\delta }{ {\mathcal L} }_{{G}_{I\to OD}}(\gamma )=\tfrac{\beta }{\beta +\delta }\frac{(\beta +\delta ){e}^{-(\beta +\delta )B}({e}^{(\beta +\delta -\gamma )B}-1)}{(\beta +\delta -\gamma )\,(1-{e}^{-(\beta +\delta )B})},\end{array}$$

so that

$${p}_{S}(INF\cap \overline{DET})=\frac{\beta }{\beta +\delta }\,[1-\frac{(\beta +\delta ){e}^{-(\beta +\delta )B}({e}^{(\beta +\delta -\gamma )B}-1)}{(\beta +\delta -\gamma )\,(1-{e}^{-(\beta +\delta )B})}].$$

We consider now some parameter values inspired from ref.^24^, that describes an outbreak of *Methicillin-resistant Staphylococcus Aureus* (*MRSA*) that took place in The London Hospital in 1982. In particular, we consider an outbreak that was 16 weeks long, where $\frac{6}{103}$ healthcare workers were infected. We translate these quantities into

$$\begin{array}{rcl}\delta  & = & \frac{1}{16}{{\rm{weeks}}}^{-1},\\ \frac{\beta }{\beta +\delta } & = & \frac{6}{103}.\end{array}$$

**Figure 12:**
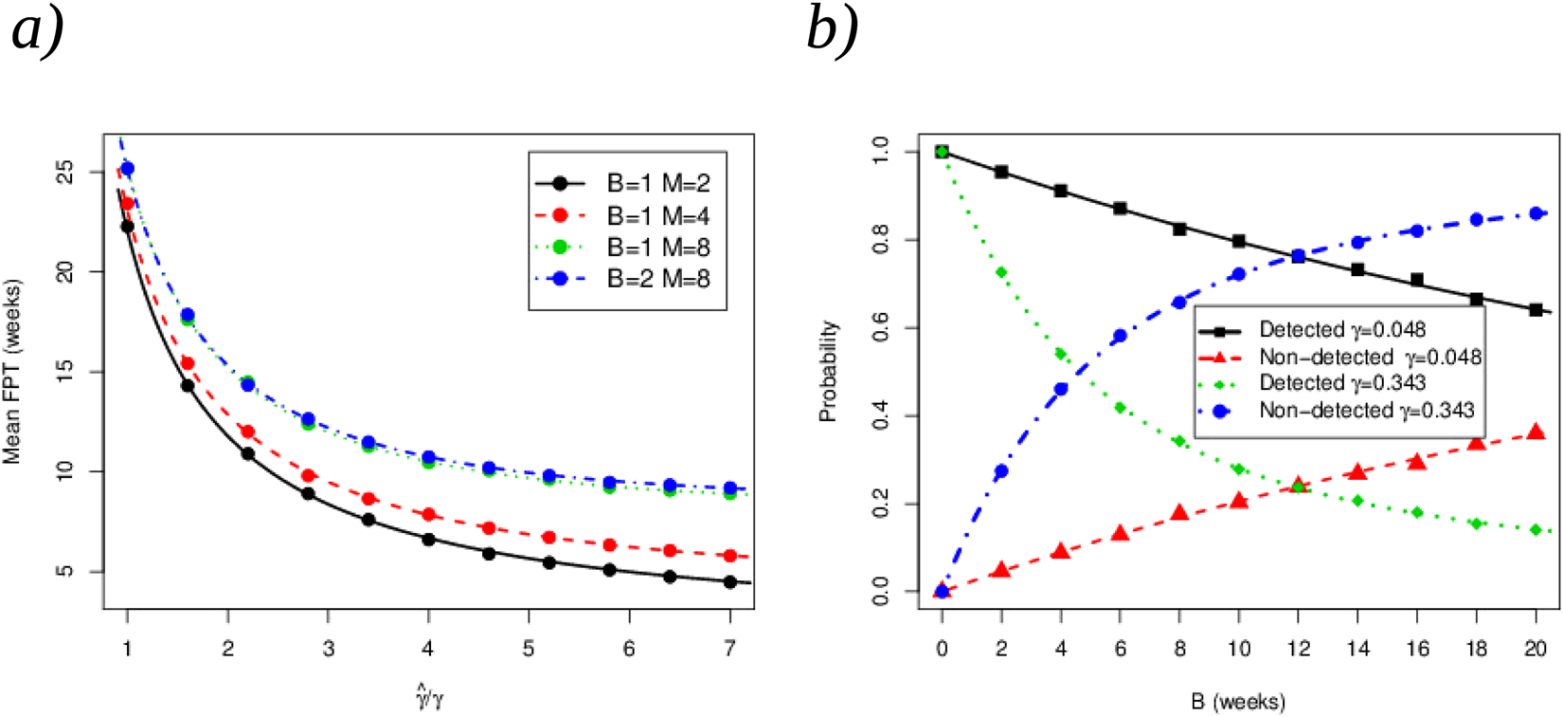
(**a**) Mean FPT to state ${\rm{\Delta }}$ from the initial infected state *I*, for different screening policies (iv) and (v) described in terms of the parameters *B* and *M*. (**b**) Probabilities of detection and non-detection for the initial state *I*.

From ref.^25^, we consider that the healthcare worker under surveillance can spontaneously recover at rate *γ* = 0.0475 weeks^−1^. In Fig. 12 we compare the theoretical prediction for the process described by Fig. 11 and numerical results from the simulation of this process. Figure 12a shows the impact of the treatment on the time to recovery if we assume that the HCW has been infected (*i*.*e*., for the initial state *I*), measured by the increase in the recovery rate after entering the off-duty state (and the subsequent treatment), $\tilde{\gamma }/\gamma$, and for different values of *B* and *M* corresponding to the screening policies in place. It can be seen that the parameter *M* (days between screening after the healthcare worker has been sent off-duty) plays a more crucial role than the screening policy in place after the outbreak is detected (related to parameter *B*), when studying the mean FPT to state ${\rm{\Delta }}$. In all cases, the symbols represent the numerical simulations and the solid lines the theoretical prediction, respectively. In Fig. 12b, we plot the probabilities of detecting and not detecting the infection for an initially infected HCW,

$${p}_{I}(INF\cap DET)\,{\rm{and}}\,{p}_{I}(INF\cap \overline{DET}),$$

for two different values of *γ* and for different values of *B*. Figure 12b shows that if the recovery occurs fast (*γ* = 0.343 weeks^−1^), a screening policy corresponding to small values of *B* (and requiring high levels of surveillance, and thus high costs) needs to be in place in order to ensure the detection of the infection for the HCW. On the other hand, detection probabilities close to one can still be obtained for relatively large values of *B* (requiring low levels of surveillance, and thus low costs) if the infectious period is large enough (*γ* = 0.048 weeks^−1^). For example, if *γ* = 0.343 weeks^−1^, the probability of the infection being undetected is near 50% for a policy corresponding to screening HCWs every *B* = 4 *weeks*. On the other hand, this screening policy allows for detecting the infection with probability above 90% if *γ* = 0.048 weeks^−1^. This example illustrates the importance of setting screening policies according to both the infectivity and the infectivity period of the specific pathogen.

## Discussion and Conclusion

In this paper, we make use of semi-Markov processes, where the absolute waiting time distribution from a given state to another is a function of the time since the system entered the first state. We have shown the relationship between the absolute waiting times to conditioned waiting times and the transition probabilities of the process. By means of first step arguments, we obtain the matrix **A**(*z*) in Eq. (1), which contains all the relevant information of the system and thus, allows for the computation of first passage times as well as the probabilities of different outcomes.

Exact computation can be carried out when all but one of the absolute waiting times for transitions (*i*.*e*., the *arrows*) out of any given state follow an exponential distribution. When more than one absolute waiting time is non-exponential, we replace all but one of the non-exponential distributions by phase-type ones. Our approach has been shown to have applications in different biological contexts. For instance, as we have illustrated in case study 1 in the Results section, we can combine different experiments to obtain molecular rates (as, for instance, the generally distributed absolute waiting time for the phosphorylation events) or, as in case study 3, the impact of different *screening* strategies on the detection or end of a nosocomial outbreak.

Our approach relies on an analytical form of the Laplace-Stieltjes transform for each of the general distributions involved in the absolute waiting times. Hence, it cannot be used when the Laplace-Stieltjes transform is not well defined (e.g., the Cauchy distribution). However, of special relevance is the use of discrete absolute waiting time distributions for which the Laplace-Stieltjes transforms are well defined, as in case study 3, or deterministic (*i*.*e*., degenerate) distributions (with ${\mathcal L} (z)={e}^{-Tz}$ and *T* a fixed but fiducial time). This might be useful in laboratory experiments where the scientist regularly interacts with the experiment (for instance, adding ligand in a binding experiment) at pre-established discrete deterministic times.

From a computational perspective, for biological systems, the matrix **A**(*z*) is usually sparse, so it is amenable for inversion algorithms that are cost-effective and thus, allows one to extend the analysis presented in our case studies to larger systems. We note that for the case studies considered in this paper, no inversion algorithms were required. For a given system represented in terms of a discrete space with *N* states, where for example two events (*i*.*e*., arrows) leaving each state are generally distributed, one can follow our approach and replace one of these generally distributed absolute waiting times, per state, by an *M*-dimensional phase-type distribution, which would lead to the analysis of an alternative stochastic process with *MN* states; similarly to our example in Fig. 9, where *M* = 3. Although the resulting **A**(*z*) might be sparse, so that efficient computational techniques could be in principle applied, computational limitations might still arise for significantly large values of *N*, when approximating more than one general distribution by a phase-type one for many events, or when high-dimensional phase-type approximations were needed (instead of the three-dimensional one used in Fig. 9, which might directly depend on the particular general distributions to be approximated). In the case of having an infinite number of states, the applicability of our approach relies on finding appropriate recursive solutions for the (infinite) system given by Eq. (1).

It is also worth mentioning that one natural generalisation of our approach is to study other quantities and not only first passage times, such as the probabilities of not becoming infected, becoming infected and being detected, or becoming infected but not being detected in case study 3), or in general any summary statistic or stochastic descriptor of the system allowing for a first step analysis approach. We note that the differences observed when studying first passage times for processes with generally distributed inter-event times, compared to their Markovian counterparts, could be even more significant when analysing different observables (stochastic descriptors). Analysing other stochastic descriptors is out of the scope of the present paper and will be the aim of our future collaborative effort.

As a final caveat, it is worth placing our approach in the context of the standard framework provided by ODE models, that are accurate as long as the probability of extinction is small, the population is large and the waiting time distributions of the underlying stochastic process are exponential. Without this latter assumption, the most accurate description is not an ordinary but an integro-differential equation (where the *kernel* of the distribution couples the value of the variables at the current time with their values in the past). Some authors have included this assumption (for instance, to model the eclipse phase in a viral infection) making use of delay differential equations^26^. As shown in Fig. 10b, replacing only one of the events by a general distribution is not accurate unless the other competing times also accommodate non-exponential distributions^27,28^.

## Electronic supplementary material

Supplementary Information

## Data Availability

Computer codes to generate the results derived in this paper are available online at Castro M, López-García M, Lythe G, Molina-París C. First passage events in biological systems with non-exponential inter-event times (computer codes). University of Leeds. [Dataset] 10.5518/359.
