# Supplementary material for: First passage events in biological systems with non-exponential inter-event times
Source: Sci Rep. 2018 Oct 10;8:15054. doi: 10.1038/s41598-018-32961-7 (PMC6180141; doi:10.1038/s41598-018-32961-7)
Supplement: Supplementary file 1 — Supplementary Information [file 41598_2018_32961_MOESM1_ESM.pdf]

Supplementary Information: First passage events in biological systems with non-exponential inter-event times

Mario Castro[a,b,*], Martín López-García[b], Grant Lythe[b], and Carmen Molina-París[b]

[a]Grupo Interdisciplinar de Sistemas Complejos (GISC) and DNL, Universidad Pontificia Comillas, Madrid E-28015, Spain
[b]Department of Applied Mathematics, School of Mathematics, University of Leeds, Leeds LS2 9JT, UK
[*]Corresponding author: marioc@comillas.edu

ABSTRACT

In this Supplementary Information we show how to compute, under the scenarios described in Fig. 3 in the main text, the probabilities, p_{ij}, and the conditioned transforms, $\mathscr{C}_{ij}(z)$, from the absolute waiting time distributions (*i.e.,* distributions of times $T_{i\to j}$, for $i, j \in \mathscr{S}$), so that Eq. (1) in the main text can be applied in order to study first passage times, $T_i(\Omega)$, or other quantities of interest (see case study 3 in Section Results in the main text). This document does not contain any figure so, all the references to Figures are those in the main text.

1 Competing events

Let us consider that the process $\mathscr{X}$ just arrived into state $i \in \mathscr{S}$ at the current time. When analysing the next event to occur, we focus on the conditioned times $T_{i\to j}|i \to j$ and the probabilities of these events taking place, $p_{ij} = \mathbb{P}(i \to j)$. In general, the probability density of these conditioned times is given by

$$f_{T_{i\to j}|i\to j}(t) = \frac{d\mathbb{P}(T_{i\to j} < t|i \to j)}{dt}\ , \quad j \in A(i)\ , \tag{1}$$

where

$$\mathbb{P}(T_{i\to j} < t|i \to j) = \frac{\mathbb{P}(T_{i\to j} \leq \min(t, T_{i\to k}\ :\ \forall k \neq j))}{\mathbb{P}(i \to j)} = \frac{1}{p_{ij}} \int_0^t dt_j\ f_{T_{i\to j}}(t_j) \prod_{k\neq j}(1 - F_{T_{i\to k}}(t_j))\ , \quad j \in A(i)\ , \tag{2}$$

and

$$p_{ij} = \mathbb{P}(i \to j) = \int_0^{+\infty} dt_j\ f_{T_{i\to j}}(t_j) \prod_{k\neq j}(1 - F_{T_{i\to k}}(t_j))\ , \quad j \in A(i)\ , \tag{3}$$

where $f_{T_{i\to j}}(t)$ is the *absolute* waiting time probability density; that is, the density of $T_{i\to j}$, and $F_{T_{i\to k}}(t)$ represents the absolute cumulative waiting time density of $T_{i\to k}$. The latter integral allows one to combine information about every transition in the absence of the others into a conditioned probability that the event $i \to j$ actually occurs before any other transition.

Eq. (1), Eq. (2) and Eq. (3) do not seem very useful when the times $T_{i\to j}$ are generally distributed as in Fig. 3*b left*. However, when all but one absolute waiting times are exponentially or phase-type distributed, as in Fig. 3*a* or Fig. 3*b right*, Eq. (1), Eq. (2) and Eq. (3) simplify and allow actual computations. Thus, in the following sections we describe how to apply these equations when, from any state $i \in \mathscr{S}$, there are n_i directly accessible states $j \in A(i) = \{i_1, i_2, \ldots, i_{n_i-1}\} \cup \{i_{n_i}\}$ with corresponding absolute waiting times $T_{i\to j}$ as in Fig. 3*a* or Fig. 3*b right* in the main text.

1.1 Probability of an exponential transition taking place before a general one

For the sake of simplicity, let us first consider the case where there are only two possible transitions out of state i ($n_i = 2$ *arrows* leaving i in Fig. 3): transition to a state i_1, occurring after an exponentially distributed random time $T_{i\to i_1} \equiv T_{Exp(\lambda_{i,i_1})} \sim Exp(\lambda_{i,i_1})$, and to a state i_2 occurring after a generally distributed random time $T_{i\to i_2} \equiv T_G \sim G$. In this case Eq. (2) above can

be written as

$$\begin{aligned}\mathbb{P}(T_{Exp(\lambda_{i,i_1})} < t | T_{Exp(\lambda_{i,i_1})} < T_G) &= \frac{1}{\mathbb{P}(T_{Exp(\lambda_{i,i_1})} < T_G)} \int_0^t dt_1\, f_{T_{Exp(\lambda_{i,i_1})}}(t_1)(1 - F_{T_G}(t_1)) \\ &= \frac{1}{\mathbb{P}(T_{Exp(\lambda_{i,i_1})} < T_G)} \int_0^t dt_1\, \lambda_{i,i_1} e^{-\lambda_{i,i_1} t_1}(1 - F_{T_G}(t_1))\,,\end{aligned} \tag{4}$$

and Eq. (3) as

$$\mathbb{P}(T_{Exp(\lambda_{i,i_1})} < T_G) = \int_0^{+\infty} dt_1\, \lambda_{i,i_1} e^{-\lambda_{i,i_1} t_1}(1 - F_{T_G}(t_1))\,. \tag{5}$$

Eq. (5) is the Laplace-Stieltjes transform of $1 - F_{T_G}(t)$ with argument $z = \lambda_{i,i_1}$ and the Laplace transform of the time derivative of Eq. (4) is also the Laplace-Stieltjes transform of $1 - F_{T_G}(t)$ with argument $z + \lambda_{i,i_1}$. Finally, we get

$$p_{i,i_1} = \mathbb{P}(i \to i_1) = \mathbb{P}(T_{Exp(\lambda_{i,i_1})} < T_G) = 1 - \mathscr{L}_G(\lambda_{i,i_1})\,, \tag{6}$$

and

$$\mathscr{C}_{i,i_1}(z) \equiv E[e^{-zT_{Exp(\lambda_{i,i_1})}} | T_{Exp(\lambda_{i,i_1})} < T_G] = \frac{\lambda_{i,i_1}}{(\lambda_{i,i_1} + z)} \frac{1 - \mathscr{L}_G(z + \lambda_{i,i_1})}{1 - \mathscr{L}_G(\lambda_{i,i_1})}\,, \tag{7}$$

where $\mathscr{L}_G(z) = E[e^{-zT_G}]$ is the Laplace-Stieltjes transform of the generally distributed waiting time $T_G \sim G$.

For the general case in which we have $n_i - 1$ exponentially distributed and one generally distributed waiting times, as in Fig. 3a, it is possible to obtain the following analogous expressions

$$\begin{aligned}p_{ij} &= \mathbb{P}(i \to j) = \mathbb{P}(T_{Exp(\lambda_{ij})} < min\{T_G, T_{Exp(\lambda_{ik})} : k \neq j\}) = \frac{\lambda_{ij}}{\sum_{k=i_1}^{i_{n_i-1}} \lambda_{ik}} \left(1 - \mathscr{L}_G\left(\sum_{k=i_1}^{i_{n_i-1}} \lambda_{ik}\right)\right)\,, \quad j \in \{i_1, i_2, \dots, i_{n_i-1}\}\,, \\ \mathscr{C}_{ij}(z) &\equiv E[e^{-zT_{Exp(\lambda_{ij})}} | T_{Exp(\lambda_{ij})} < min\{T_G, T_{Exp(\lambda_{ik})} : k \neq j\}] = \frac{\sum_{k=i_1}^{i_{n_i-1}} \lambda_{ik}}{\sum_{k=i_1}^{i_{n_i-1}} \lambda_{ik} + z} \frac{1 - \mathscr{L}_G\left(z + \sum_{k=i_1}^{i_{n_i-1}} \lambda_{ik}\right)}{1 - \mathscr{L}_G\left(\sum_{k=i_1}^{i_{n_i-1}} \lambda_{ik}\right)}\,, \quad j \in \{i_1, i_2, \dots, i_{n_i-1}\}\,.\end{aligned}$$

1.2 Probability of a general transition taking place before an exponential one

In a similar fashion, and when considering $n_i = 2$ in Fig. 3a, we can analyse the probability of $i \to i_2$ taking place before $i \to i_1$. In particular, it is easy to see that

$$p_{i,i_2} = P(T_G < T_{Exp(\lambda_{i,i_1})}) = \mathscr{L}_G(\lambda_{i,i_1})\,, \tag{8}$$

and

$$\mathscr{C}_{i,i_2}(z) \equiv E[e^{-zT_G} | T_G < T_{Exp(\lambda_{i,i_1})}] = \frac{\mathscr{L}_G(z + \lambda_{i,i_1})}{\mathscr{L}_G(\lambda_{i,i_1})}\,. \tag{9}$$

We note that we can also work in *backward* fashion. That is, making use of the properties of functional equations[1], we can invert Eq. (9) to obtain the absolute waiting time distribution as a function of the conditioned one. In particular, we find

$$\mathscr{L}_G(z) = \frac{\mathscr{C}_{i,i_2}(z - \lambda_{i,i_1})}{\mathscr{C}_{i,i_2}(-\lambda_{i,i_1})}\,. \tag{10}$$

This result is striking as it allows one to obtain information about the absolute waiting time distribution of an event from the observation of the distribution conditioned that it has been experimentally observed. In fact, by the properties of the Laplace-Stieltjes transform, Eq. (10) can be transformed back to the time domain, leading to the density function

$$f_{T_G}(t) = \frac{1}{\mathscr{C}_{i,i_2}(-\lambda_{i,i_1})} e^{-\lambda_{i,i_1} t} f_{T_G | T_G < T_{Exp(\lambda_{i,i_1})}}(t)\,, \tag{11}$$

where $\mathscr{C}_{i,i_2}(-\lambda_{i,i_1})$ is a normalisation constant.

Finally, similar formulae can be obtained for a general number n_i of states in Fig. 3*a*. In particular, we get Eq. (2) in the main text, namely

$$p_{i,i_{n_i}} = \mathbb{P}(i \to i_{n_i}) = \mathbb{P}(T_G < min\{T_{Exp(\lambda_{ij})}: j \in \{i_1, i_2, \ldots, i_{n_i-1}\}\}) = \mathscr{L}_G\left(\sum_{j=i_1}^{i_{n_i-1}} \lambda_{ij}\right),$$

$$\mathscr{C}_{i,i_{n_i}}(z) = \frac{\mathscr{L}_G\left(z + \sum_{j=i_1}^{i_{n_i-1}} \lambda_{ij}\right)}{\mathscr{L}_G\left(\sum_{j=i_1}^{i_{n_i-1}} \lambda_{ij}\right)}.$$

1.3 Generalisation to the case of one phase-type waiting time and a general one

The arguments in the previous sections relate to a generally distributed waiting time competing with several exponentially distributed waiting times. It is possible to generalise these arguments for phase-type distributions instead of exponential ones. This can be extremely useful, given that the family of phase-type (PH) distributions is dense in the family of non-negative continuous distributions, and there exist algorithms to approximate any generally distributed random variable by a phase-type random variable[2]. This means that in Fig. 3*b left*, when trying to consider more than one generally distributed waiting time, we could replace all but one generally distributed waiting times by phase-type distributions, as in Fig. 3*b right*, and then our approach would also apply.

A phase-type distribution is defined as the time to absorption for a continuous time Markov chain with infinitesimal generator of the form

$$\begin{pmatrix} \mathbf{T} & \mathbf{T}^0 \\ \mathbf{0} & 0 \end{pmatrix},$$

and it can be seen as a generalisation of the exponential distribution (see Chapter 1 in[2]). The density function of a phase-type distribution $PH(\alpha, \mathbf{T})$ is given by

$$f_{PH}(t) = \alpha \cdot e^{\mathbf{T}t} \cdot \mathbf{T}^0, \tag{12}$$

where $\mathbf{T}^0 \equiv -\mathbf{T} \cdot \mathbf{e}$, $\mathbf{e}$ is a column vector of 1's, and α is the vector of initial probabilities.

Let us consider, without loss of generality, $n_i = 2$ in Fig. 3*b right*: from state i the system moves to state i_1 after a time $T_{i \to i_1} \equiv T_{PH}$ that follows a phase-type distribution, and moves to state i_2 after a generally distributed time $T_{i \to i_2} \equiv T_G$. We note that our arguments can be easily generalised to larger values of n_i, where $n_i - 1$ phase-type distributed times *compete* against a generally distributed time T_G. The distribution function of this phase-type distribution can be expressed as[3]

$$F_{T_{PH}}(t) = \mathbb{P}(T_{PH} < t) = 1 - \sum_{i=1}^{N} p_i(t) e^{-\mu_i t}$$

where N is the number of eigenvalues of $\mathbf{T}$, $p_i(t)$ is a polynomial of order $L_i - 1$,

$$p_i(t) = \sum_{j=0}^{L_i-1} a_{ij} t^j, \quad i \in \{1, \ldots, N\},$$

and L_i is the multiplicity of the eigenvalue $-\mu_i$. It is clear that the survival function and the density function of this phase-type random variable are given by

$$S_{T_{PH}}(t) = \mathbb{P}(T_{PH} > t) = \sum_{i=1}^{N} p_i(t) e^{-\mu_i t},$$

$$f_{T_{PH}}(t) = \frac{d}{dt} F_{T_{PH}}(t) = \sum_{i=1}^{N} (\mu_i p_i(t) - p_i'(t)) e^{-\mu_i t} = \sum_{i=1}^{N} q_i(t) e^{-\mu_i t},$$

where

$$q_i(t) = \sum_{j=0}^{L_i-1} b_{ij} t^j, \quad i \in \{1, \ldots, N\},$$

with

$$\begin{aligned} b_{ij} &= \mu_i a_{ij} - (j+1)a_{ij+1}, \quad j = 0,\ldots,L_i - 2, \\ b_{iL_i-1} &= \mu_i a_{iL_i-1}, \end{aligned}$$

for all $i \in \{1,\ldots,N\}$. One can compute first the conditioned density function

$$f_{T_G|T_G<T_{PH}}(t) = \frac{1}{\mathbb{P}(T_G < T_{PH})} f_{T_G}(t)(1 - F_{T_{PH}}(t)) = \frac{1}{\mathbb{P}(T_G < T_{PH})} f_{T_G}(t) \sum_{i=1}^{N} p_i(t) e^{-\mu_i t},$$

so that the conditioned transform is given by

$$\begin{aligned} \mathscr{C}_{i,i_2}(z) &= E[e^{-zT_G} \mid T_G < T_{PH}] = \frac{1}{\mathbb{P}(T_G < T_{PH})} \int_0^{+\infty} e^{-zt} f_{T_G}(t) \sum_{i=1}^{N} p_i(t) e^{-\mu_i t} dt = \frac{1}{\mathbb{P}(T_G < T_{PH})} \sum_{i=1}^{N} \sum_{j=0}^{L_i-1} a_{ij} \int_0^{+\infty} f_{T_G}(t) t^j e^{-(z+\mu_i)t} dt \\ &= \frac{1}{\mathbb{P}(T_G < T_{PH})} \sum_{i=1}^{N} \sum_{j=0}^{L_i-1} a_{ij} (-1)^j \frac{d^j}{dz^j} \mathscr{L}_G(z+\mu_i). \end{aligned}$$

The probability $\mathbb{P}(T_G < T_{PH})$ can be obtained as

$$p_{i,i_2} = \mathbb{P}(T_G < T_{PH}) = \int_0^{+\infty} dt_1 f_{T_G}(t_1) \int_{t_1}^{+\infty} f_{T_{PH}}(t_2) dt_2 = \sum_{i=1}^{N} \sum_{j=0}^{L_i-1} a_{ij} \int_0^{+\infty} f_{T_G}(t_1) t_1^j e^{-\mu_i t_1} dt_1 = \sum_{i=1}^{N} \sum_{j=0}^{L_i-1} a_{ij} (-1)^j \frac{d^j}{dz^j} \mathscr{L}_G(z) \bigg|_{z=\mu_i}.$$

It is clear that

$$p_{i,i_1} = \mathbb{P}(T_{PH} < T_G) = 1 - p_{i,i_2} = 1 - \sum_{i=1}^{N} \sum_{j=0}^{L_i-1} a_{ij} (-1)^j \frac{d^j}{dz^j} \mathscr{L}_G(z) \bigg|_{z=\mu_i},$$

and that

$$f_{T_{PH}|T_{PH}<T_G}(t) = \frac{1}{\mathbb{P}(T_{PH} < T_G)} f_{T_{PH}}(t)(1 - F_{T_G}(t)) = \frac{1}{\mathbb{P}(T_{PH} < T_G)} (1 - F_{T_G}(t)) \sum_{i=1}^{N} \sum_{j=0}^{L_i-1} b_{ij} t^j e^{-\mu_i t}.$$

Then, the conditioned Laplace-Sieltjes transform can be obtained by following similar arguments than above, and is given by

$$\mathscr{C}_{i,i_1}(z) = E[e^{-zT_{PH}} \mid T_{PH} < T_G] = \frac{1}{\mathbb{P}(T_{PH} < T_G)} \sum_{i=1}^{N} \sum_{j=0}^{L_i-1} b_{ij} (-1)^j \frac{d^j}{dz^j} \mathscr{V}_G(z+\mu_i),$$

where $\mathscr{V}(z)$ is the Laplace transform of the survival function of the general distribution,

$$\mathscr{V}_G(z) = \frac{1 - \mathscr{L}_G(z)}{z}.$$

We finally note that, if the phase-type distribution under analysis corresponds to a diagonalisable matrix $\mathbf{T}$, which is usually the case when the PhaseType R package[4] is used for approximating a general distribution (see case study 2 and the corresponding phase-type distribution in Fig. 9 in the main text), and the density function can be expressed as

$$f_{T_{PH}}(t) = \sum_{i=1}^{N} c_i \mu_i e^{-\mu_i t}.$$

In this way, expressions above simplify as follows

$$\begin{aligned} p_{i,i_1} &= \mathbb{P}(T_{PH} < T_G) = 1 - \sum_{i=1}^{N} c_i \mathscr{L}_G(\mu_i), \quad p_{i,i_2} = \sum_{i=1}^{N} c_i \mathscr{L}_G(\mu_i), \\ \mathscr{C}_{i,i_1}(z) &= E[e^{-zT_{PH}} | T_{PH} < T_G] = \sum_{i=1}^{N} c_i \frac{\mu_i}{z+\mu_i} \frac{1 - \mathscr{L}_G(z+\mu_i)}{1 - \mathscr{L}_G(\mu_i)}, \quad \mathscr{C}_{i,i_2}(z) = \frac{\sum_{i=1}^{N} c_i \mathscr{L}_G(z+\mu_i)}{\sum_{i=1}^{N} c_i \mathscr{L}_G(\mu_i)}. \end{aligned}$$

References

1. Castillo, E. *Functional equations and modelling in science and engineering*, vol. 161 (CRC Press, 1992).
2. He, Q.-M. *Fundamentals of matrix-analytic methods* (Springer, 2014).
3. Horvath, A., Scarpa, M. & Telek, M. Phase type and matrix exponential distributions in stochastic modeling. In *Principles of Performance and Reliability Modeling and Evaluation*, 3–25 (Springer, 2016).
4. Aslett, L. J. & Wilson, S. P. Markov chain monte carlo for inference on phase-type models. *Int. Statistical Inst.: Proc. 58th World Statistical Congress, 2011, Dublin (Session CPS027)* (2011).
